# microRNA Profile of High-Grade B-Cell Lymphoma with 11q Aberration

**DOI:** 10.3390/ijms26010285

**Published:** 2024-12-31

**Authors:** Michalina Zajdel, Łukasz Michał Szafron, Agnieszka Paziewska, Grzegorz Rymkiewicz, Michalina Dąbrowska, Zbigniew Bystydzieński, Mariusz Kulińczak, Beata Grygalewicz, Maria Sromek, Katarzyna Błachnio, Maria Kulecka, Filip Hajdyła, Krzysztof Goryca, Magdalena Chechlińska, Jan Konrad Siwicki

**Affiliations:** 1 Department of Cancer Biology, Maria Sklodowska-Curie National Research Institute of Oncology, Roentgena 5, 02-781 Warszawa, Polandfilip.hajdyla@nio.gov.pl (F.H.); magdalena.chechlinska@nio.gov.pl (M.Ch.); 2 Department of Genetics, Maria Sklodowska-Curie National Research Institute of Oncology, Roentgena 5, 02-781 Warszawa, Poland; lukszafron@gmail.com (Ł.M.S.);; 3 Faculty of Medical and Health Sciences, Siedlce University, Konarskiego 2, 08-110 Siedlce, Poland; 4 Flow Cytometry Laboratory, Department of Cancer Pathomorphology, Maria Sklodowska-Curie National Research Institute of Oncology, Roentgena 5, 02-781 Warszawa, Poland; 5 Cytogentics Laboratory, Maria Sklodowska-Curie National Research Institute of Oncology, Roentgena 5, 02-781 Warszawa, Poland

**Keywords:** HGBCL-11q, BL, GCB-DLBCL-NOS, microRNA, NGS

## Abstract

High-grade B-cell lymphoma with 11q aberration (HGBCL-11q) is a rare germi-nal centre lymphoma characterised by a typical gain/loss pattern on chromo-some 11q but without MYC translocation. It shares some features with Burkitt lymphoma (BL), HGBCLs and germinal centre-derived diffuse large B-cell lym-phoma, not otherwise specified (GCB-DLBCL-NOS). Since microRNA expression in HGBCL-11q remains unknown, we aimed to identify and compare the mi-croRNA expression profiles in HGBCL-11q, BL and in GCB-DLBCL-NOS. Next-generation sequencing (NGS)-based microRNA profiling of HGBCL-11q (*n* = 6), BL (*n* = 8), and GCB-DLBCL-NOS without (*n* = 3) and with MYC rearrange-ment (MYC-R) (*n* = 7) was performed. We identified sets of 39, 64, and 49 mi-croRNAs differentiating HGBCL-11q from BL, and from GCB-DLBCL-NOS without MYC-R, respectively. The expression levels of miR-223-3p, miR-193b-3p, miR-29b-3p, and miR-146a-5p consistently differentiated HGBCL-11q from both BL, GCB-DLBCL-NOS without MYC-R. In addition, HGBCL-11q presented greater heterogeneity in microRNA expression than BL. The expression profile of MYC-regulated microRNAs differed in HGBCL-11q and in BL, while also clearly distinguishing HGBCL-11q and BL from GCB-DLBCL-NOS. The microRNA pro-file of HGBCL-11q differs from those of BL and GCB-DLBCL-NOS, exhibiting greater heterogeneity compared to BL. The microRNA profile further supports that HGBCL-11q is a distinct subtype of B-cell lymphoma.

## 1. Introduction

The recent 5th edition of the World Health Organization Classification of Haematolymphoid Tumours: Lymphoid Neoplasms (WHO-HAEM5) changed the name of the provisional entity “Burkitt-like lymphoma with 11q aberration” from the 4th edition to “High-grade B-cell lymphoma with 11q aberration” (HGBCL-11q) [1,2]. This lymphoma is also included in the latest classification of the International Consensus Committee (ICC 2022) as a provisional entity named large B-cell lymphoma with 11q aberration [3]. HGBCL-11q is very rare [4]; it accounts for only about 3–10% of Burkitt lymphoma (BL) diagnoses [5,6,7,8,9]. HGBCL-11q is of germinal centre origin (as BL and germinal centre-derived diffuse large B-cell lymphoma, not otherwise specified, GCB-DLBCL-NOS), and carries typical aberrations in the long arm of chromosome 11, characterised by a simultaneous gain of the 11q23.2-23.3 region, often with its inversion, and by the 11q24.1-qter loss, yet it lacks *MYC* rearrangement (*MYC*-R) [10,11,12]. The presence of 11q24.1-qter telomeric loss or its substitute—the loss of heterozygosity—is essential for diagnosing HGBCL-11q [2,4].

The morphology of HGBCL-11q is similar to that of BL, often featuring a starry sky appearance formed by coarse apoptotic debris infiltrated by macrophages, as well as intermediate or blastoid morphology [2,13].

The immunophenotype of HGBCL-11q, detected by immunohistochemistry (IHC) and flow cytometry (FCM), is similar to that of BL with a few exceptions. In FCM studies, differences in immunophenotype between HGBCL-11q and BL are particularly evident [14]. HGBCL-11q also shows a good response to the treatment regimens typical for BL [14].

An early study by Pienkowska-Grela et al. [10] described the recurrent dup(11)(q23q13) aberration in a group of lymphomas with a morphology similar or identical to that of BL but lacking *MYC*-R and supposed that the 11q abnormality may relate to the phenotype similarity of the two entities. Moreover, Ferreiro et al. [15] found that post-transplant and immunocompromised molecularly defined BL (mBL) are frequently MYC-negative and display an 11q/-gain/loss pattern. Their analyses of three cases of mBL with 11q/-gain/loss aberration showed that the genes harbouring minimal gained and minimal lost regions were implicated, among others, with *MYC* networks, and suggested that the 11q-gain/loss aberration is a “molecular variant” of t(8q24/*MYC*) [15].

The level of MYC expression in HGBCL-11q may vary, and despite the absence of 11q cases frequently show nodal presentation and more complex karyotypes than BL, which typically show extranodal presentation and simple karyotypes. A significant proportion of HGBCL-11q cases also present molecular features typical of germinal centre-derived diffuse large B-cell lymphoma, not otherwise specified (GCB-DLBCL-NOS), or of high-grade B-cell lymphoma, not otherwise specified (HGBCL-NOS) [12,16,17,18]. The mutational landscape of HGBCL-11q was defined as closer to GCB-DLBCL-NOS than to BL [19]. Another study demonstrated that the genomic and mutational profiles of HGBCL-11q are more similar to that of HGBCL or GCB-DLBCL than of BL [16]. Most reports described HGBCL-11q as a primary disease; still, Woroniecka et al. reported on the case of HGBCL-11q as a Richter transformation [20].

The deregulation of mRNA expression by microRNA has been suggested as an important mechanism in the pathogenesis of lymphoma. For example, the integrative analysis through the correlation of microRNA and mRNA profiles revealed defined miRNA/mRNA target interactions in BL, DLBCL, and follicular lymphoma, and demonstrated that microRNA expression changes contributed to the deregulated expression of many genes and associated regulatory pathways with important roles in lymphomagenesis [21]. Moreover, many authors suggest that microRNA expression profiling could be a valuable component of diagnostic algorithms for B-cell non-Hodgkin lymphomas (B-NHLs) [22,23,24,25,26,27,28]. However, the microRNA expression profile of HGBCL-11q has not yet been studied. To fill this gap, we performed a next-generation sequencing (NGS)-based profiling of microRNA expression in HGBCL-11q, BL, and GCB-DLBCL-NOS with and without *MYC*-R.

Our study showed that the HGBCL-11q microRNA expression profile is different and more heterogeneous than that of BL and also different from that of GCB-DLBCL-NOS cases. We also identified four microRNAs that differentiated HGBCL-11q from BL as well as from GCB-DLBCL-NOS without *MYC*-R. It further underscores that HGBCL-11q represents a molecularly distinct subtype of B-cell lymphoma.

## 2. Results

### 2.1. NGS Analysis of microRNA Expression Profiles of HGBCL-11q Compared to BL

The expression levels of 39 microRNAs differed significantly between the analysed groups, with 33 microRNAs showing significantly higher and 6—lower expression in HGBCL-11q compared to BL (Table 1).

The results of the NGS were validated in HGBCL-11q and BL by RT-qPCR against the selected reference microRNAs (Table S6 and Figure S7).

**Figure 1:**
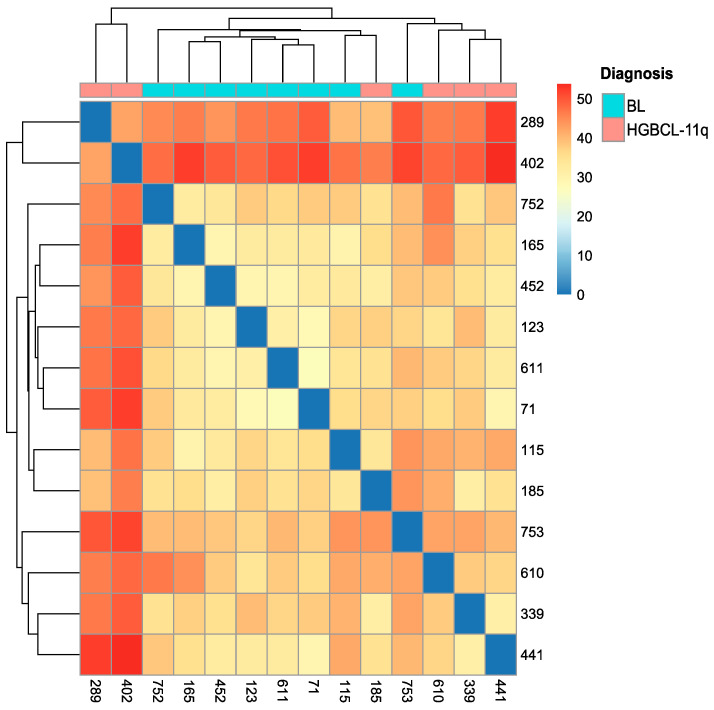
Dendrogram and heatmap illustrating the hierarchical clustering of the HGBCL-11q and BL samples based on the expression levels of all the microRNA NGS reads for each individual sample. This analysis highlights the similarities and differences in the microRNA expression levels across individual samples. The colours, as indicated by the scale, represent differences in expression profiles, ranging from none (dark blue) to high (dark red).

Unsupervised hierarchical clustering based on the expression levels of all microRNA NGS reads revealed greater variability in the microRNA expression profile within HGBCL-11q compared to BL (Figure 1). Two HGBCL-11q samples, 289 and 402, exhibited significantly distinct microRNA expression profiles, differing both from the rest of the HGBCL-11q group and from BL. Conversely, one HGBCL-11q sample (nr 185) showed a microRNA expression profile more similar to the BL group, while one BL sample (nr 753) showed a microRNA expression profile closer to HGBCL-11q.

The hierarchical clustering of HGBCL-11q and BL samples is based on the expression levels of all the microRNAs read in NGS analysis.

**Figure 2:**
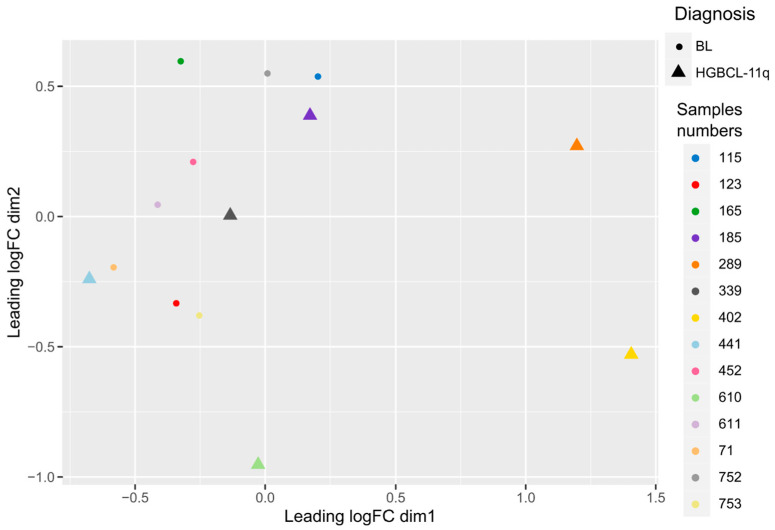
MDS analysis performed for the HGBCL-11q and BL cases based on the expression profiles of NGS reads for all microRNAs. dim1 and dim2 represent the first and second dimensions, equivalents of the first and second principal components in the PCA. The X and Y axes—measure of the diversity of each principal component.

Multidimensional scaling (MDS), an equivalent of principal component analysis, revealed the similarities of the microRNA expression profiles in three HGBCL-11q and in the BL cases. However, three other HGBCL-11q samples (nr: 289, 402, and 610) showed different microRNA expression profiles (Figure 2).

The MDS analysis and hierarchical clustering both showed greater heterogeneity in the microRNA profiles in HGBCL-11q as compared to the more homogenous profiles in BL.

Multidimensional scaling analysis of HGBCL-11q and BL microRNA profiles.

### 2.2. MicroRNA Expression Profiles Differentiating HGBCL-11q and BL in the Context of Cytogenetic Changes

**Figure 3:**
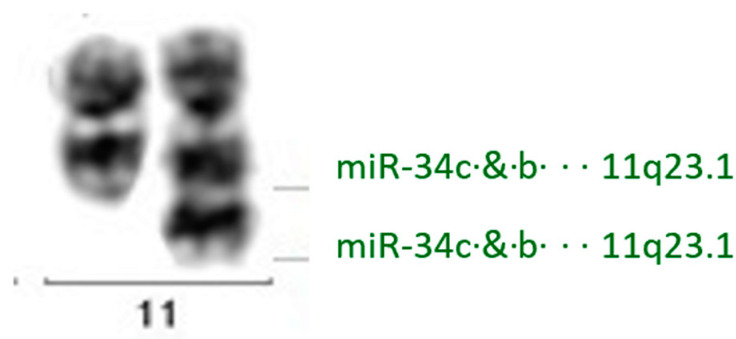
The pair of chromosomes 11 in HGBCL-11q with a duplication at locus 11q23.1 on the long arm of one of the chromosomes.

Our cytogenetic data revealed DNA duplication at locus 11q23.1 in all the HGBCL-11q cases (Table 2 and Figure 3). This locus contains genes for two molecules within a single cluster: miR-34b (chr11: 111512938-111513021 [+] forward strand, ID in Ensembl Database ENSG00000207811; GRCh38:CM000673.2) and miR-34c (chr11: 111513439-111513515 [+] forward strand, ID in Ensembl Database ENSG00000207562; GRCh38:CM000673.2). Notably, our NGS analysis showed a statistically significant increase in the expression levels of miR-34b-5p (*FC* = 4.056; padj = 0.0436) and miR-34c-5p (*FC* = 5.63; padj *=* 0.0068) in HGBCL-11q compared to BL.

### 2.3. NGS Analysis of MicroRNA Expression Profiles in HGBCL-11q Compared to GCB-DLBCL-NOS Without and with MYC-R

We analysed and compared the microRNA expression levels in HGBCL-11q (*n* = 6) and GCB-DLBCL-NOS without *MYC*-R (*n* = 3) due to the postulated similarity of these entities, and also to GCB-DLBCL-NOS with *MYC*-R (*n* = 6) to indirectly measure the impact of *MYC* rearrangement on microRNA profile expression. The expression levels of 64 microRNAs differed significantly between HGBCL-11q and GCB-DLBCL-NOS without *MYC*-R. The expression levels of 49 microRNAs differ from HGBCL-11q and GCB-DLBCL-NOS with *MYC*-R. In Supplementary Materials we present a list of microRNAs whose expression levels significantly differentiated HGBCL-11q and both subgroups of GCB-DLBCL-NOS (Tables S1 and S2)

The hierarchical clustering analysis and MDS analysis were then performed for HGBCL-11q and two subgroups of GCB-DLBCL-NOS. It revealed distinct microRNA profiles between HGBCL-11q and the above-mentioned two subgroups of GCB-DLBCL-NOS, but a low number of GCB-DLBCL-NOS without *MYC*-R limits further conclusions (Figures S1–S4).

Subsequently, we have compared the lists of the microRNAs expressed in the studied samples, and we identified four microRNAs whose expression differentiated HGBCL-11q from both GCB-DLBCL-NOS without *MYC*-R and also BL: miR-223-3p, miR-193b-3p, miR-29b-3p, and miR-146a-5p (these microRNAs are highlighted bold green in Table 1, Tables S1 and S2). Moreover, the expression levels of miR-223-3p and miR-193b-3p also differentiated HGBCL-11q from GCB-DLBCL-NOS with *MYC*-R. The expression levels of miR-223-3p and miR-193b-3p were lower in HGBCL-11q than in the other groups studied. The expression of miR-29b-3p was higher in HGBCL-11q than in both GCB-DLBCL-NOS without *MYC*-R and BL. The expression of miR-146a-5p was higher in HGBCL-11q than in the BL cases, but lower in HGBCL-11q than in GCB-DLBCL-NOS without *MYC*-R. But it should be noted that our results are based on a low number of cases and should be confirmed on larger sets of samples (Table 3).

### 2.4. Expression Profile of MYC-Regulated microRNAs in HGBCL-11q, BL, and GCB-DLBCL-NOS with and Without MYC Rearrangement

Given the important role of MYC transcription factor in B-NHLs, particu-larly in BL, and the suggestion that 11q aberration may induce biological effects similar to MYC rearrangement, we also focused on MYC-regulated microRNAs: miR-15a-5p, miR-17-5p, miR-17-3p, miR-18a-5p, miR-18a-3p, miR-19a-3p, miR-19b-1-5p, miR-20a-5p, miR-20a-3p, miR-20b-5p, miR-23a-3p, miR-23b-3p, miR-25-5p, miR-26a-5p, miR-29b-3p, miR-29c-5p, miR-30e-5p, miR-30e-3p, miR-34a-5p, miR-92a-1-5p, miR-93-3p, miR-106b-5p, miR-106b-3p, miR-130b-3p, miR-142-3p, miR-146a-5p, miR-150-5p, miR-150-3p, miR-155-5p, miR-184, and miR-196b-5p [25,29,30,31,32,33,34,35].

**Figure 4:**
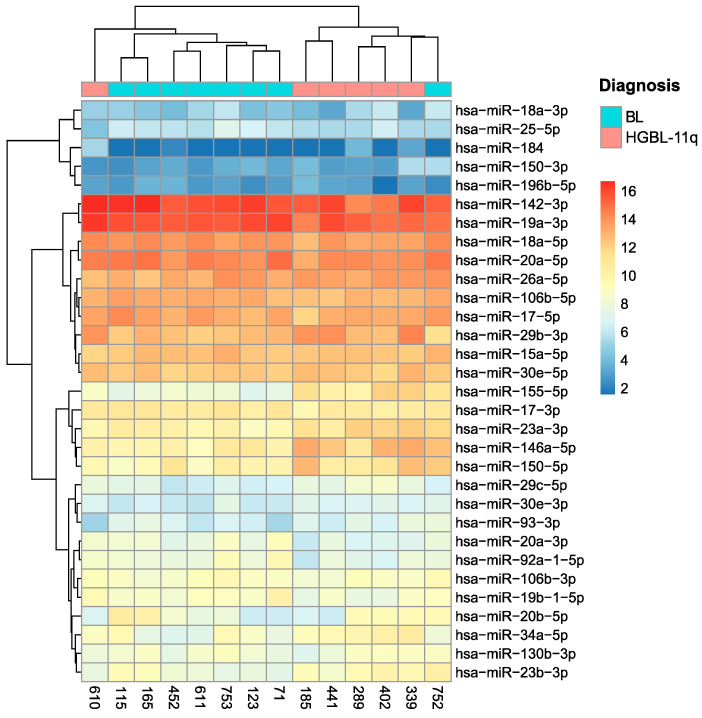
Dendrogram and heatmap illustrating the hierarchical clustering of the HGBCL-11q and BL samples based on the expression levels of MYC-regulated microRNAs. The colours, as indicated by the scale, represent differences in expression levels, ranging from none (dark blue) to high (dark red).

**Figure 5:**
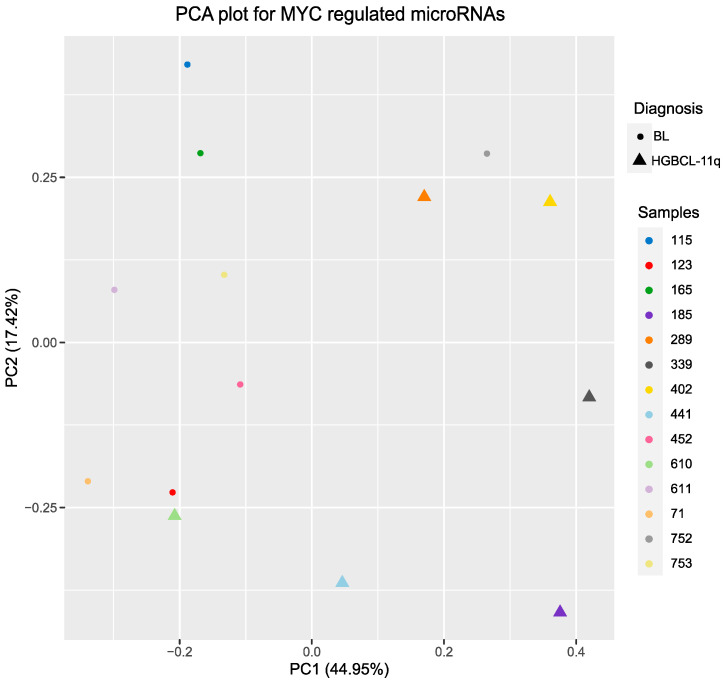
PCA performed for the HGBCL-11q and BL cases based on the expression profiles of NGS reads for the MYC-regulated microRNAs. PC1 and PC2 represent the first and second principal components. The X and Y axes are measures of the diversity of each principal component.

First, we analysed the expression levels of MYC-regulated microRNAs in BL and HGBCL-11q. Both the hierarchical clustering and PCA for the MYC-regulated microRNAs revealed a clear separation between the HGBCL-11q and BL samples. However, two samples, one from HGBCL-11q and one from BL, demonstrated a similarity of microRNAs regulated by MYC to the opposite group (Figure 4 and Figure 5).

**Figure 6:**
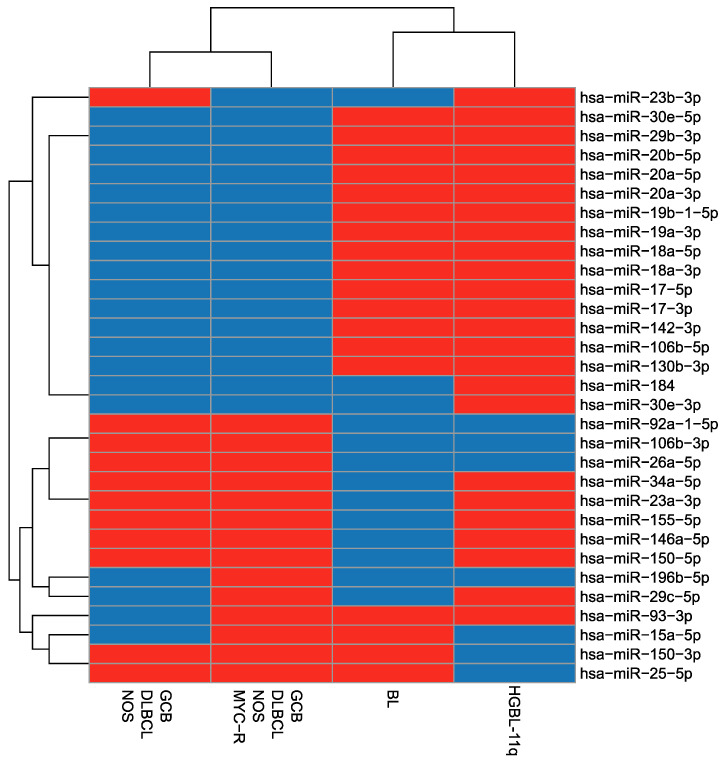
Dendrogram and heatmap illustrating hierarchical clustering of the HGBCL-11q, BL GCB-DLBCL-NOS without *MYC*-R, and GCB-DLBCL-NOS with *MYC*-R samples based on the categorising of the expression levels of the MYC-regulated microRNAs. Dark blue colour indicates that the mean microRNA expression in a given group is lower than the median expression of that microRNA across all samples; red indicates that the mean microRNA expression in a given group is higher than or equal to the median expression of that microRNA across all the samples.

Next, we performed the analysis of MYC-regulated microRNAs including also GCB-DLBCL-NOS with and without MYC-R. That analysis indicated that the profile of MYC-regulated microRNAs is most similar to the profile observed for BL. Moreover, a clear distinction of BL and HGBCL-11q from GCB-DLBCL-NOS was observed (Figure 6).

The categorisation of the MYC-regulated microRNA expression levels ena-bled the comparison of the relative expression proportions of these microRNAs across the B-NHL groups in relation to the BL group.

This analysis revealed the following:

- A 61.3% similarity between the HGBCL-11q group and the BL group (19 out of 31 microRNAs presenting the same microRNA pattern);
- A 19.4% similarity between the GCB-DLBCL-NOS group and the BL group (6 out of 31 microRNAs presenting the same microRNA pattern);
- A 22.6% similarity between the GCB-DLBCL-NOS with the *MYC*-R group and the BL group (7 out of 31 microRNAs presenting the same microRNA pattern).

The analysis of the MYC-regulated microRNAs indicated that among the four lymphoma types, HGBCL-11q showed the greatest similarity to BL. At the same time, clear differences in the expression levels of MYC-regulated mi-croRNAs were observed between the HGBCL-11q and BL groups.

### 2.5. Ontological Analyses

Ontological analyses were carried out using the miEAA programme and: 1/ Reactome and miRPathDB databases; 2/ GeneOntology and miRPathDB data-bases. The first analysis, performed on the group of microRNA whose expression differentiated HGBCL-11q and BL, showed that these molecules are involved in regulating a variety of signalling pathways and/or biological processes. Among the most strongly associated signalling pathways were those related to signal transduction processes involving interleukins (IL-4, IL-6, IL-10, and IL-13) (Tables S7 and S8). The second analysis identifying molecular functions connected with microRNAs that differentiate HGBCL-11q and BL revealed 109 molecular functions, including: cytokine receptor binding, signalling receptor binding, and signalling receptor activator activity (Table S9).

Another analysis performed for microRNAs that differentiate HGBCL-11q and BL using data from the analysis of the interaction of microRNAs with the transcripts of their target genes revealed that these microRNAs are involved in the regulation of 298 biological processes, with B lymphocyte proliferation and arginine catabolic processes being the most prominent. This analysis was con-ducted using miEAA and databases: Gene Ontology and miRTarBase (Table S10).

## 3. Discussion

The microRNA profile of HGBCL-11q is still not characterised. We have previously shown differences in the expression of miR-155, its precursor *BIC*, miR-21, and miR-26a between DLBCL-NOS and BL, and indicated similar expression patterns of these microRNAs and *BIC* in HGBCL-11q and BL, suggesting a close ontogenetic relationship between HGBCL-11q and BL [25].

Here, we show that the microRNA profile of HGBCL-11q differs from that of BL and GCB-DLBCL-NOS. There were significantly different expression levels of 39 microRNAs between HGBCL-11q and BL. The ontological analyses linked those differentially expressed microRNAs, among other pathways and processes, with microenvironment properties, including signal transduction processes involving interleukins and cytokine receptor binding. We observed higher microRNA profile heterogeneity in HGBCL-11q than in classical BL. Di Stefano et al., 2022 have recently suggested heterogeneity of HGBCL-11q based on mRNA expression [36]. We also found that the expression levels of 64 microRNA significantly differ between HGBCL-11q and GCB-DLBCL-NOS without *MYC*-R, and 49 microRNA differ between HGBCL-11q and GCB-DLBCL-NOS with *MYC*-R.

Given the significance of *MYC*-R in the pathogenesis of aggressive B-NHLs and in microRNA biogenesis, we also analysed the expression patterns of microRNAs potentially regulated by MYC. We found that the profile of MYC-regulated microRNAs differentiates HGBCL-11q and BL. However, it is worth mentioning that HGBCL-11q showed the highest similarity to BL when GCB-DLBCL-NOS, both with and without *MYC*-R, were included in the analysis.

In addition, we found that the expression levels of miR-146a-5p, miR-29b-3p, miR-223-3p, and miR-193-b-3p differentiate HGBCL-11q from both BL and GCB-DLBCL-NOS without *MYC*-R. We observed that the expression of miR-223-3p and miR-193-b-3p was decreased in HGBCL-11q as compared to BL and to GCB-DLBCL-NOS, both with and without *MYC*-R. miR-223 is involved in the regulation of lymphocyte B differentiation and can act as an oncogene, as seen in e.g., T-cell acute lymphoblastic leukaemia, or as a tumour suppressor in acute myeloid leukaemia (AML) [37]. Decreased expression of miR-223 has been observed in various types of lymphomas and chronic lymphocytic leukaemia (CLL) and was associated with an adverse prognosis [38,39]. The role of miR-193b-3p in the development of B-NHLs is almost unknown. However, similar to miR-223, decreased expression of miR-193b-3p was observed in CLL compared to healthy individuals [40] and in AML [41]. Previous studies have demonstrated the important tumour-suppressive role of miR-193b-3p, as it targets several members of the RAS-RAF-MEK-ERK pathway which is crucial for cell cycle and proliferation, as well as c-KIT [41,42] and the *MYB* oncogene [43].

We also observed increased expression of miR-29b-3p in HGBCL-11q compared to BL and GCB-DLBCL-NOS without *MYC*-R, but it did not differentiate HGBCL-11q from GCB-DLBCL-NOS with *MYC*-R. The members of the miR-29 family, like many other microRNAs, can function as tumour suppressors or as oncomiRs. However, the majority of the literature data suggests their antiapoptotic functions [44]. Their target genes comprise *BCL2* and *MCL1* (from the *BCL2* family), which code for antiapoptotic proteins, as well as the protooncogene *B-MYB* [44]. miR-29b-3p is negatively regulated by the MYC transcription factor, and decreased expression of miR-29 was observed in BL in comparison to DLBCL, CLL, mantle cell lymphoma (MCL), and follicular lymphoma (FL) without *MYC* translocation [23,26,30]. These findings highlight the complexity of the pathological mechanisms underlying aggressive lymphomas and are in line with the statement by the Loeffler-Wirth group that, on a molecular level, mature B-NHLs do not clearly separate, but instead form a continuum of expression patterns [45].

We also observed that the expression level of miR-146a-5p is higher in HGBL-11q than in BL, but lower than in GCB-DLBCL-NOS without *MYC*-R. There was no difference in the expression level between HGBCL-11q and GCB-DLBCL-NOS with *MYC*-R. The expression of miR-146a-5p is negatively regulated by the MYC transcription factor and has been found to differentiate BL and DLBCL [21,26]. We also observed that the expression level of miR-146a-5p significantly and strongly differentiates BL from GCB-DLBCL-NOS without *MYC*-R. In our analyses, the expression level of this microRNA also significantly differentiates BL from GCB-DLBCL-NOS with *MYC*-R, although the difference is smaller than in comparison with GCB-DLBCL-NOS without *MYC*-R. This difference likely reflects the extent of negative MYC regulation in BL and GCB-DLBCL-NOS with *MYC* rearrangement.

The analysis of the microRNA profile differentiating HGBCL-11q and BL in the context of the obtained cytogenetic data showed that the significantly increased expression of miR-34b-5p and of miR-34c-5p in HGBCL-11q compared to BL coincided with DNA duplication at locus 11q23.1 (present in all our HGBCL-11q cases), where both microRNAs are located. This suggests that the DNA duplication at this locus may be the mechanism responsible for the higher expression of the two microRNAs in HGBCL-11q.

A limitation of our study is the small number of cases, and confirming the relevance of the identified profiles will require further studies with larger groups of specimens.

In summary, our study indicates that the microRNA profile of HGBCL-11q, including the expression pattern of MYC-regulated microRNAs, differs from that of both BL and GCB-DLBCL-NOS. The expression levels of miR-223-3p, miR-193b-3p, miR-29b-3p, and miR-146a-5p differentiate HGBCL-11q from both BL and GCB-DLBCL-NOS without *MYC*-R. The microRNA profile further confirms HGBCL11q as a distinct subtype of B-cell lymphoma.

## 4. Materials and Methods

### 4.1. Patients and Samples

The study included cell aspirate samples obtained prospectively by fine-needle aspiration biopsy (FNAB) collected from 24 patients diagnosed and treated at the Department of Lymphoid Malignancies, Maria Sklodowska-Curie National Research Institute of Oncology in Warsaw in the years 2006–2017: 6 HGBCL-11q, 8 BL, 3 GCB-DLBCL-NOS without *MYC*-R, and 7 GCB-DLBCL-NOS with *MYC*-R. The study was conducted in accordance with the Declaration of Helsinki, and the protocol was approved by the Ethics Committee of the Maria Sklodowska-Curie National Research Institute of Oncology (4/2011/1/2012; 17 April 2012).

The final diagnoses of all the B-NHL cases were based on histopathological (HP) examinations with the immunohistochemistry (IHC) of surgically collected material and a flow cytometric (FCM) analysis of the FNAB collected material. In our hospital, for all the patients with clinical- or HP/IHC-suspected HGBCL-11q, BL, DLBCL-NOS, and other aggressive lymphomas, FNAB is performed for FCM, classical cytogenetics (CC) including karyotyping and FISH, and molecular biology studies, including array-based comparative genomic hybridisation (aCGH) and next-generation sequencing (NGS). The quantitative and qualitative evaluation of lymphoma cells and subpopulations of lymphoid and macrophage cells in the suspension obtained by FNAB in FCM provides detailed information about the quality of the remaining suspension for cytogenetic–molecular analyses.

FCM of the cellular suspension obtained by the FNAB or by ultrasound-guided FNAB (in cases with bulky abdominal mass, stomach, intestine and abdominal lymph node involvement) of the involved lymph nodes, tonsils, and extranodal tumours was performed by a hematopathologist (GR—a co-author of this manuscript), who simultaneously diagnosed the same cases using HP/IHC as previously described [14].

The final diagnoses were established according to the 2016 revision of the WHO Classification of Lymphomas, WHO-HAEM5 [1,2,3,46], and our practical FCM and IHC-based approach to the diagnosis of HGBCL-11q and BL [14]. The FNAB/FCM method is the preferred diagnostic tool, and the detection of CD56 with the absence of CD38 + higher expression on lymphoma cells by means of the FCM procedure appears to be a reliable, fast, and easy method for diagnosing 11q aberrations in HGBCL-11q without *MYC* rearrangement. FCM enables the differentiation of HGBCL-11q from BL with 100% sensitivity. The diagnoses were based on the clinical characteristics of patients (rapidly growing large tumour mass, higher LDH and β-microglobulins value, and B symptoms including high fever, weight loss, and night sweats) along with extended diagnostics tools, including cytopathological (stained by the HE and MGG methods) and HP examinations, and immunophenotype assessment by IHC/FCM and CC, in accordance with the WHO-HAEM5 diagnostic recommendations [2,4].

The karyotype analysis followed standard procedures. Chromosomes were G/C-banded using Wright stain. The karyotypes were classified according to the International System for Human Cytogenetic Nomenclature [47] (Table 2—for HGBCL-11q cases and Table S3—for BL and GCB-DLBCL-NOS cases). The *MYC* gene rearrangement was assessed by the FISH technique. The FISH examination was performed using probes specific for genes in the duplicated region: *CCND1* (11q13.3), *ATM* (11q22.3), *KMT2A* (11q23.3), and using *D11S1037* (11q25) probe, which is located in the region of terminal deletion. Aberrations were determined by calculating the ratio of signals from the above-mentioned probes to the signal from the chromosome 11 centromeric probe (D11Z1). Gene rearrangements were also tested for the: *BCL2*, *BCL6*. Samples with additional gene rearrangements were excluded from the microRNA expression analysis. Catalogue number, company name, and probe characteristics are summarised in Table S5 in Supplementary Material.

For the HGBCL-11q samples, array comparative genomic hybridisation (aCGH) was also applied.

All the HGBCL-11q samples were characterised according to the algorithm presented in our previous publication [14]. All the HGBCL-11q samples were EBV-negative. Confirmed viral infection with HIV, HCV, or HBV was an exclusion criterion. The clinico-pathological features cases with HGBCL-11q, BL, and GCB-DLBCL-NOS are summarised in Table 4. The immunophenotype of the cases analysed is presented in Table S4.

### 4.2. Next-Generation Sequencing (NGS)

Total RNA was isolated from snap-frozen FNAB-acquired cells using TriReagent (cat no. AM9738, ThermoFisher Scientific, Waltham, MA USA) and stored at –70 °C. RNA purity and concentration were measured using a NanoDrop ND-1000 spectrophotometer (ThermoFisher Scientific). The concentration of microRNA in the total RNA was estimated in an Agilent 2100 Bioanalyser with the use of the Agilent Small RNA Kit (cat. no. 5067-1548, Agilent Technologies, Santa Clara, CA, USA). Sequencing was performed on an Ion Proton sequencer (Thermo Fisher Scientific). MicroRNA libraries were prepared using the Ion Total RNA-seq Kit v2 for Small RNA Libraries (cat. no. 4479789, Thermo Fisher Scientific) and Ion Xpress™ RNA-Seq Barcode 01-16 Kit (cat. no. 4475485, Thermo Fisher Scientific). Template preparation for sequencing was performed on an Ion Chef instrument (Thermo Fisher Scientific) using the Ion PI™ Hi-Q™ Chef Kit reagents (cat. no. A26772, Thermo Fisher Scientific) and the Ion PI™ Chip Kit v3 (cat. no. A26771, Thermo Fisher Scientific). All the procedures were conducted according to the manufacturer’s protocol.

The data presented in this publication have been deposited in NCBI’s Gene Expression Omnibus (GEO) and are accessible through GEO Series accession number GSE281999 at https://www.ncbi.nlm.nih.gov/geo/query/acc.cgi?acc=GSE281999 accessed on 1 June 2025.

### 4.3. Bioinformatics

The raw sequencing results were analysed in Torrent Suite (version 5.6), including sequence reading, truncation of adapter sequences, and assignment of reads to the correct samples. Unmapped bam files were converted to fastq files using the bamToFastq script from the BEDTools package (version 2.225) [48]. NGS reads were mapped to the human genome (hg19) using the BWA software version 0.7.17. Reads for known, i.e., included in the miRBase database (version 21), and new microRNAs were counted using the miRDeep2 application (version 2.0.1.2).

The data normalisation step and the assessment of changes in microRNA expression between the analysed lymphomas were performed in edgeR (version 3.20.6) [49]. The statistical significance of differences in NGS read counts between the studied groups was evaluated using the likelihood ratio test with false discovery rate (FDR) correction according to Benjamini and Hochberg [50].

Ontology analyses for microRNAs, the expression of which differentiated the studied lymphomas, were performed using the microRNA Enrichment Analysis and Annotation tool (miEAA; version 2.0) [51] and the following databases: Gene Ontology, Reactome, miRPathDB, and miRTarBase.

Bioinformatics and statistical analyses, including the hierarchical clustering of the results and the assessment of expression for the MYC-dependent microRNAs, were carried out in the R environment (version 3.6.1.). The NGS data for the individual samples were also compared by multidimensional scaling (MDS) using the limma package (version 3.42.20) [29] and by performing the principal component analysis (PCA) applying the plotPCA function from the DESeq2 package (version 1.26.0).

### 4.4. Verification of the MicroRNA NGS Results Performed on HGBCL-11q and BL Groups

The NGS data were verified in HGBCL-11q and BL by assessing the expression levels of miR-155-5p (ID 000479), miR-21-5p (ID 000397), miR-29b-2-5p (ID 002166), miR-29b-3p (ID 000413), miR-200c-3p (ID 002300), miR-223-3p (ID 002295), and miR-1295a (ID 002862) (specific primers and probe sets cat. no. 4427975, Thermo Fisher Scientific) using RT-qPCR and the ∆CT calculation method.

To select reference microRNAs for validations using qPCR, the first criterion was the stability of microRNA expression in the NGS data (NormFinder version 5 in R). Additional criteria were a high number of reads in the NGS analysis, a high fold change in the expression level between HGBCL-11q and BL as well as its statistical significance, and supporting evidence from the literature.

The arithmetic mean of the expression levels of five microRNAs was used as the reference expression level: miR-26a-5p (ID 000405), miR-148b-3p (ID 000471), miR-103a-3p (ID 000439), miR-185-5p (ID 002271), and miR-26b-5p (ID 000406) (specific primers and probe sets cat. no. 4427975, Thermo Fisher Scientific), as each of those microRNAs met the criteria of the NormFinder algorithm.

### 4.5. Basic Statistical Analyses and Basic Graphic Illustrations

The obtained results were mostly not normally distributed, as assessed by the Shapiro–Wilk normality test. Therefore, differences in the microRNA levels between the HGBCL-11q and BL groups revealed by the RT-qPCR were evaluated using the nonparametric Mann–Whitney test. Differences were considered statistically significant at *p* < 0.05. Basic statistical analyses and the graphic illustrations of the results were performed using GraphPad (versions 6 and 8)

## Figures and Tables

**Table 1:** microRNAs significantly differentiating HGBCL-11q and BL.

microRNA	*p*	*p* After *FDR* Correction *	Fold Change in the Expression Level (FC **) HGBCL-11q vs. BL	Absolute FC Value—Measure of Effect Size Between HGBCL-11q and BL (The Direction of Change Is Not Determined)
**Downregulated**				
miR-1295a	1.02 × 10^−10^	5.29 × 10^−8^	**0.0089**	112.6981
miR-1258	0.0015691	0.0479931	**0.0986**	10.1412
miR-374b-3p	0.0004055	0.0234316	**0.1135**	8.8086
miR-4464	0.0001835	0.0132941	**0.1364**	7.3324
**^ miR-193b-3p**	**0.0017997**	**0.0479931**	**0.168**	**5.954**
**^ miR-223-3p**	**7.96 × 10^−5^**	**0.0075223**	**0.2368**	**4.2232**
**Upregulated**				
miR-375	5.26 × 10^−6^	0.0010935	**411.1885**	411.1885
miR-217	0.0005125	0.0266517	**116.0331**	116.0331
miR-3919	1.11 × 10^−5^	0.0019193	**82.7087**	82.7087
miR-203a-3p	0.000789	0.0356757	**21.3876**	21.3876
miR-184	0.0013256	0.0475389	**19.0457**	19.0457
miR-3922-5p	0.0001566	0.0125251	**18.7733**	18.7733
miR-1273f	0.0002085	0.0135519	**16.1614**	16.1614
miR-663a	8.91 × 10^−5^	0.0077217	**14.1112**	14.1112
miR-3648	0.0015867	0.0479931	**12.6628**	12.6628
miR-663b	0.0012033	0.0446922	**11.7792**	11.7792
miR-3687	7.07 × 10^−5^	0.0073518	**11.6062**	11.6062
miR-5585-3p	5.54 × 10^−5^	0.0072047	**10.6479**	10.6479
miR-200c-3p	3.48 × 10^−6^	0.000905	**9.7307**	9.7307
miR-1908-5p	0.0007598	0.0356757	**9.4687**	9.4687
miR-4423-3p	0.0001917	0.0132941	**9.4671**	9.4671
miR-5096	0.0004547	0.0248916	**8.4225**	8.4225
miR-619-5p	6.97 × 10^−5^	0.0073518	**7.3868**	7.3868
miR-21-3p	4.25 × 10^−7^	0.0001473	**6.837**	6.837
miR-21-5p	3.68 × 10^−18^	3.83 × 10^−15^	**6.3138**	6.3138
miR-3681-3p	0.001682	0.0479931	**5.7853**	5.7853
miR-34c-5p	4.61 × 10^−5^	0.0068419	**5.6338**	5.6338
miR-146a-3p	0.0011608	0.0446922	**5.6252**	5.6252
miR-2116-5p	0.0014039	0.0479931	**5.6157**	5.6157
miR-155-5p	0.001047	0.0435563	**4.6529**	4.6529
miR-34a-3p	0.0017452	0.0479931	**4.4065**	4.4065
miR-221-5p	0.0017977	0.0479931	**4.0981**	4.0981
miR-34b-5p	0.0010133	0.0435563	**4.0561**	4.0561
miR-28-3p	0.0011707	0.0446922	**3.573**	3.573
miR-29b-2-5p	0.0003843	0.0234316	**3.4938**	3.4938
**miR-146a-5p**	**0.0015166**	**0.0479931**	**3.456**	**3.456**
miR-28-5p	0.0014925	0.0479931	**3.3087**	3.3087
**miR-29b-3p**	**0.0006006**	**0.0297432**	**2.4175**	**2.4175**
miR-30d-5p	0.001781	0.0479931	**2.1034**	2.1034

* FDR—false discovery rate; ** FC—fold-change; values below 1—decrease in expression; values above 1—increase in expression; bold—indicates microRNAs whose expression levels differ HGBCL-11q, BL, and also GCB-DLBCL-NOS without *MYC*-R; ^ bold—indicates microRNAs whose expression levels differ HGBCL-11q, BL, GCB-DLBCL-NOS without *MYC*-R, and GCB-DLBCL-NOS with *MYC*-R.

**Table 2:** Cytogenetic data of HGBCL-11q cases.

No.	Karyotype [Standard GC-Banding—Wright Stain] and FISH	Array Comparative Genomic Hybridisation (aCGH) [hg19]	Rearrengment		
			*MYC*	*BCL2*	*BCL6*
**610**	46,XY,dup(11)(q23.1q13.1). nuc ish (MYCx2) [238], (BCL6x2) [255], (BCL2x2) [252]	arr[hg19] 11q13.1q23.3(65,526,674_120,557,653)x3 11q23.3q25(120,392,484_134,931,948)x1	(-)	(-)	(-)
**339**	45,X,-Y, del(6)(q21), dup(11)(q24.3q12.1). nuc ish (MYCx2) [247], (BCL6x2) [200], (BCL2x2) [207]	arr[hg19] 11q12.1q24.3(56,790,631_128,177,729)x3 11q24.3q25(128,177,670_134,931,948)x1	(-)	(-)	(-)
**289**	46,XY,dup(11)(q24.1q22.3) [11]. nuc ish (MYCx2) [153], (BCL6x2) [154], (BCL2x2) [187]	arr[hg19] 11q22.3q24.1(106,120,397_123,495,005)x3, 11q23.3q24.1(118,239,916_123,495,005)x4, 11q24.1q25(123,572,602_134,931,948)x1, 11q24.3 (128,039,399_128,813,918)x0	(-)	(-)	(-)
**185.1**	42~45,X,-Y,-4,add(4)(q12), der(6)t(4;6)(q12;p25), dup(11)(q14q24), dup(11)(q24q14), +1~2mar[cp11]/44~45, idem, +der(11)(11pter->11q24::11q14->11q24::?) [cp6]. nuc ish (MYCx2) [247], (BCL6x2) [318], (BCL2x2) [267]	arr[hg19]11q14.1q24.1(80,201,232_ 121,236,822)x3, 11q22.3q23.3(107,196,633_120,744,339)x4, 11q24.1q25(121,335,329_134,586,308)x2	(-)	(-)	(-)
**441**	46,XY,?del(3)(q2?7), del(6)(q2?2), dup(11)(q24.1q13.1), der(18)t(3;18)(q2?7;q21). nuc ish (MYCx2) [124], (BCL6x3) [120/157], (BCL2x1) [105/139]	arr [hg19] 11q13.1-24.1(63,708,104_121,434,719)x3, 11q24.1-q25(121,434,660_134,931,948)x1	(-)	(-) 1 copy in 76%	(-) 3 copies in 76%
**402**	46,XY, dup(11)(q23.3q22.2)/47, sl, +12/48, sdl1, +3. nuc ish (MYCx2) [137], (BCL6x3) [38/216], (BCL2x2) [200]	arr[hg19] 11q22.2q23.3 (102,144,117_121,118,244)x3, 11q23.3 (117,815,640_119,275,901)x4, 11q24.1q25 (121,346,328_134,931,948)x1	(-)	(-)	(-) 3 copies in 18%

No.—sample number; aCGH—array-based comparative genomic hybridisation; add—additional material of unknown origin; cp—composite karyotype; del—deletion; der—derivative chromosome; dup—duplication; FISH—fluorescence in situ hybridisation; hg—human genome version 19; idem—denotes the stemline karyotype in a subclone; nuc ish—nuclear in situ hybridisation; mar—marker chromosome; pter—terminal end of the short arm; sdl—sideline; sl—stemline.

**Table 3:** microRNAs whose expressions differentiate HGBCL-11q from GCB-DLBCL-NOS without/with *MYC*-R and BL (preliminary results).

	HGBCL-11q vs. BL		HGBCL-11q vs. GCB-DLBCL-NOS without *MYC-R*		HGBCL-11q vs. GCB-DLBCL-NOS with *MYC-R*	
miR-223-3p	DECREASED IN HGBCL-11q *FC* = 0.2368 *p*_adj_ = 0.0075	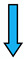	DECREASED IN HGBCL-11q *FC* = 0.0349 *p*_adj_ = 5.63 × 10^−6^	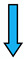	DECREASED IN HGBCL-11q *FC* = 0.0720 *p*_adj_ = 0.0024	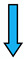
miR-193b-3p	DECREASED IN HGBCL-11q *FC* = 0.1680 *p*_adj_ = 0.0479	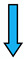	DECREASED IN HGBCL-11q *FC* = 0.0661 *p*_adj_ = 0.000675	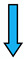	DECREASED IN HGBCL-11q *FC* = 0.0703 *p*_adj_ = 0.0370	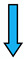
miR-29b-3p	INCREASED IN HGBCL-11q *FC* = 2.4175 *p*_adj_ = 0.0297	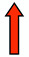	INCREASED IN HGBCL-11q *FC* = 6.0680 *p*_adj_ = 0.00355	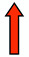	Does not differentiate	
miR-146a-5p	INCREASED IN HGBCL-11q *FC* = 3.4560 *p*_adj_ = 0.0479	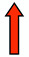	DECREASED IN HGBCL-11q *FC* = 0.0349 *p*_adj_ = 5.94 × 10^−5^	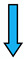	Does not differentiate	

Blue arrow—decrease expression in HGBCL-11q; red arrow—increase expression in HGBCL-11q.

**Table 4:** Clinical data of HGBCL-11q, BL, and GCB-DLBCL-NOS cases.

No.	Age/Sex	Site of Involvement	Nodal (N) vs. Extranodal (EN)	PS	CS	B	BM/CSF	LDH > UNV	IPI	Treatment	EBV
**HGBCL-11q**											
**610**	18/M	LNc	N	1	I	no	no/no	no	1	before	neg
**339**	23/M	T/LNc	EN/N	0	I	no	no/no	no	0	before	neg
**289**	40/M	LNab	N	1	IVB	yes	no/no	yes	2	before	neg
**185.1**	29/M	LNc	N	0	I	no	no/no	no	0	before	neg
**441**	32/M	T	EN	1	I	no	no/no	no	0	before	neg
**402**	62/M	LNab	N	1	IVA	no	no/no	yes	3	before	neg
**BL**											
**165**	61/F	LNc	N	0	IIIA	no	no/no	no	3	before	pos
**71**	17/M	LNc	N	0	IIIA	no	no/no	no	1	before	neg
**123**	25/M	LNc	N	0	IA	no	no/no	no	0	before	neg
**115**	39/M	ab	EN	0	IVA	no	no/no	no	2	before	neg
**452**	62/F	LNc	N	0	IA	no	no/no	yes	2	before	pos
**611**	28/M	ab	EN	3/4	IVB	yes	no/no	no	3	before	neg
**752**	36/F	Th	EN	0	IVB	yes	no/nd	no	2	before	nd
**753**	21/M	LNax/ab	EN/N	1	IVA	no	no/no	no	2	before	pos
**GCB-DLBCL-NOS without *MYC*-R**											
**758**	59/F	LNsub/LNc	EX/N	1	IIIB	yes	no/no	yes	2	before	neg
**762**	54/F	LNsub/T	EX/N	1	IVB	yes	nd/nd	no	2	before	neg
**763**	41/M	LNc	N	0	IA	no	nd/no	no	0	before	nd
	**GCB-DLBCL-NOS with *MYC*-R**										
**466**	33/F	soft tissue/leg	EN	2	IVB	yes	no/no	yes	4	before	neg
**351**	59/M	LN ^2^/ab/thor	N	1	IVB	yes	no/no	yes	3	before	nd
**751**	80/F	LNsub/soft tissue/cheek	EN/N	1	IIB	yes	nd/no	yes	2	before	neg
**755**	64/F	soft tissue/cheek	EN	1	IE	no	nd/no	no	1	before	nd
**1263**	66/M	LNing	N	0	IIA	no	no/nd	no	1	before	neg
**302**	76/F	Th	EN	0	IVA	no	no/nd	yes	3	before	nd
**35**	69/M	LNc	N	0	IA	no	no/nd	no	1	before	neg

No.—sample number; M—male; LN—lymph node; ^2^—a few neighbouring enlarged LN/tumours; ab—abdominal; ax—axillary; c—cervical; ing—inguinal; T—tonsil; Th—thyroid; thor—thorax; sub—submandibular; PS—performance status; CS—Ann Arbor Stage of disease; B—B symptoms; BM—bone marrow involvement; CSF—cerebrospinal fluid involvement; LDH > UNV—lactate dehydrogenase elevated above the upper normal value; IPI—international prognostic index score; nd—not performed; neg—negative; pos—positive.

## Data Availability

The data presented in this publication have been deposited in NCBI’s Gene Expression Omnibus (GEO) and are accessible through GEO Series accession number GSE281999 at https://www.ncbi.nlm.nih.gov/geo/query/acc.cgi?acc=GSE281999.

## Supplementary Materials

The following supporting information can be downloaded at https://www.mdpi.com/article/10.3390/ijms26010285/s1.
