# Supplementary material for: microRNA Profile of High-Grade B-Cell Lymphoma with 11q Aberration
Source: Int J Mol Sci. 2024 Dec 31;26(1):285. doi: 10.3390/ijms26010285 (PMC11720131; doi:10.3390/ijms26010285)
Supplement: Supplementary file 1 [file ijms-26-00285-s001.zip › ijms-3360577-supplementary.pdf]

Supplement 1

Table S1. microRNAs that expression levels significantly differentiate HGBCL-11q and GCB DLBCL,NOS without *MYC* rearrangement

microRNA	*p*	*p* after *FDR* correction*	**FC** HGBCL-11q vs. GCB-DLBCL-NOS without *MYC*-R**	Absolute FC value – measure of effect size between HGBCL-11q and. GCB-DLBCL-NOS without *MYC*-R (the direction of change is not determined)
**Downregulated**				
miR-520g-3p	4.28 x 10^{-8}<br>~~E-08~~	1.19 x 10^{-5}	**0.0061**	164.0268
miR-550a-3p	6.35 x 10^{-7}<br>~~E-07~~	5.94 x 10^{-5}	**0.0061**	164.0268
miR-520h	6.96 x 10^{-8}<br>~~E-08~~	1.46 x 10^{-5}	**0.0067**	149.4545
miR-4524a-5p	6.54 x 10^{-7}<br>~~E-07~~	5.94 x 10^{-5}	**0.0067**	149.4545
miR-518a-3p	8.79 x 10^{-7}<br>~~E-07~~	6.70 x 10^{-5}	**0.0074**	135.3755
miR-527	9.76 x 10^{-6}<br>~~E-06~~	0.000476	**0.0117**	85.2701
miR-518a-5p	1.02x 10^{-5}<br>~~E-05~~	0.000476	**0.0117**	85.2274
miR-3591-3p	0.000934	0.016306	**0.015**	66.6726
miR-519d-3p	1.16 x 10^{-5}<br>~~E-05~~	0.00051	**0.0168**	59.6578
miR-127-3p	0.000136	0.003451	**0.0169**	59.0882
miR-4524a-3p	1.06 x $^{10\ -8}$<br>~~E-08~~	5.63 x 10^{-6}	**0.0171**	58.3648
miR-518e-3p	2.25 x 10^{-7}<br>~~E-07~~	3.14 x 10^{-5}	**0.0171**	58.3648
miR-3653-5p	5.43 x 10^{-5}<br>~~E-05~~	0.00175	**0.0196**	50.9028
miR-517b-3p	5.08 x 10^{-5}<br>~~E-05~~	0.001748	**0.0205**	48.8437
miR-517a-3p	5.22 x 10^{-5}<br>~~E-05~~	0.001748	**0.0205**	48.8216
miR-570-5p	0.000537	0.009871	**0.0219**	45.7107
miR-548ai	0.000542	0.009871	**0.0219**	45.7314
miR-615-3p	2.65 x 10^{-6}<br>~~E-06~~	0.000158	**0.022**	45.4463
miR-3198	0.001218	0.019632	**0.0222**	45.0205
miR-1306-5p	0.000129	0.003451	**0.0262**	38.1501
miR-193b-5p	4.37 x 10^{-5}<br>E-05	0.00162	**0.0322**	31.0357
**^miR-223-3p**	**1.34 x 10^{-8}**<br>**E-08**	**5.63** x 10^{-6}	**0.0349**	**28.6473**
**miR-146a-5p**	4.99 x 10^{-7}<br>E-07	5.94 x 10^{-5}	**0.0349**	**28.6473**
miR-146b-3p	0.000269	0.006095	**0.0366**	27.3058
miR-363-5p	0.000284	0.006105	**0.041**	24.3924
miR-494-3p	8.48 x 10^{-5}<br>E-05	0.002538	**0.0439**	22.7949

miR-326	6.09 x 10^{-6} E-06	0.00034	**0.0455**	21.9866
miR-409-3p	0.001468	0.022367	**0.0463**	21.5899
miR-532-3p	1.74 x 10^{-6} E-06	0.000112	**0.0466**	21.4677
miR-424-3p	0.001809	0.027064	**0.0635**	15.7454
**^miR-193b-3p**	**1.61 x 10^{-5} E-05**	**0.000675**	**0.0661**	**15.1285**
miR-3615	0.000368	0.007169	**0.0708**	14.1206
miR-1180-3p	0.001076	0.018037	**0.0866**	11.5537
miR-708-5p	0.000366	0.007169	**0.0927**	10.7933
miR-574-5p	0.000112	0.003124	**0.0943**	10.6017
miR-193a-5p	0.000343	0.007009	**0.1085**	9.2187
miR-92a-1-5p	0.000136	0.003451	**0.1091**	9.1666
miR-361-3p	1.23 x 10^{-6} E-06	8.56 x 10^{-5} ~~E-05~~	**0.1107**	9.0302
miR-4521	0.001928	0.028349	**0.1145**	8.7357
miR-378i	0.000103	0.002973	**0.1254**	7.976
miR-345-5p	0.001062	0.018037	**0.1309**	7.6379
miR-450a-5p	0.003317	0.043433	**0.1346**	7.4285
let-7b-5p	0.000173	0.00406	**0.1384**	7.2277
miR-500a-3p	0.002724	0.037415	**0.144**	6.9459
miR-320a	2.31 x 10^{-5} E-05	0.000922	**0.1576**	6.3453
miR-766-3p	0.00326	0.043358	**0.2225**	4.494
miR-484	0.000593	0.01057	**0.2325**	4.302
miR-423-3p	0.001319	0.020467	**0.2436**	4.1057
miR-423-5p	0.002475	0.035146	**0.2866**	3.4896
let-7a-5p	0.000313	0.006567	**0.304**	3.289
miR-191-5p	0.002349	0.033938	**0.3101**	3.225
miR-26a-5p	0.002972	0.040168	**0.3728**	2.6823
**Upregulated**				
miR-590-3p	0.000276	0.006095	**833.3333**	833.3333
miR-142-3p	1.61 x 10^{-7} E-07	2.69 x 10^{-}	**13.1926**	13.1926
miR-144-3p	0.001282	0.020268	**10.627**	10.627
miR-18a-5p	7.95 x 10^{-6} E-06	0.000416	**9.6432**	9.6432
miR-301a-3p	0.002594	0.036225	**9.2678**	9.2678
miR-29c-3p	0.001111	0.018251	**8.7032**	8.7032
miR-101-3p	0.000174	0.00406	**7.9808**	7.9808
miR-19a-3p	4.45 x 10^{-5} E-05	0.00162	**7.2674**	7.2674
**miR-29b-3p**	**0.000144**	**0.003546**	**6.068**	**6.068**
miR-30e-5p	0.00038	0.00723	**5.7737**	5.7737
miR-19b-3p	7.09 x 10^{-7} E-07	5.94 x 10^{-5}	**3.9541**	3.9541
miR-16-5p	6.62 x 10^{-5} E-05	0.002053	**3.4376**	3.4376

- *FDR – false discovery rate
- **FC – fold change; values below 1 - decrease of expression; values above 1 - increase of expression;
- bold green –microRNAs that expression levels differ HGBCL-11q, BL and GCB-DLBCL-NOS without *MYC*-R

- ^ bold green –microRNAs that expression levels differ HGBCL-11q, BL, GCB-DLBCL-NOS and also GCB-DLBCL-NOS with *MYC*-R

Hierarchical clustering of HGBCL-11q and GCB-DLBCL-NOS without *MYC*-R samples based on expression levels of all microRNAs read in NGS analysis

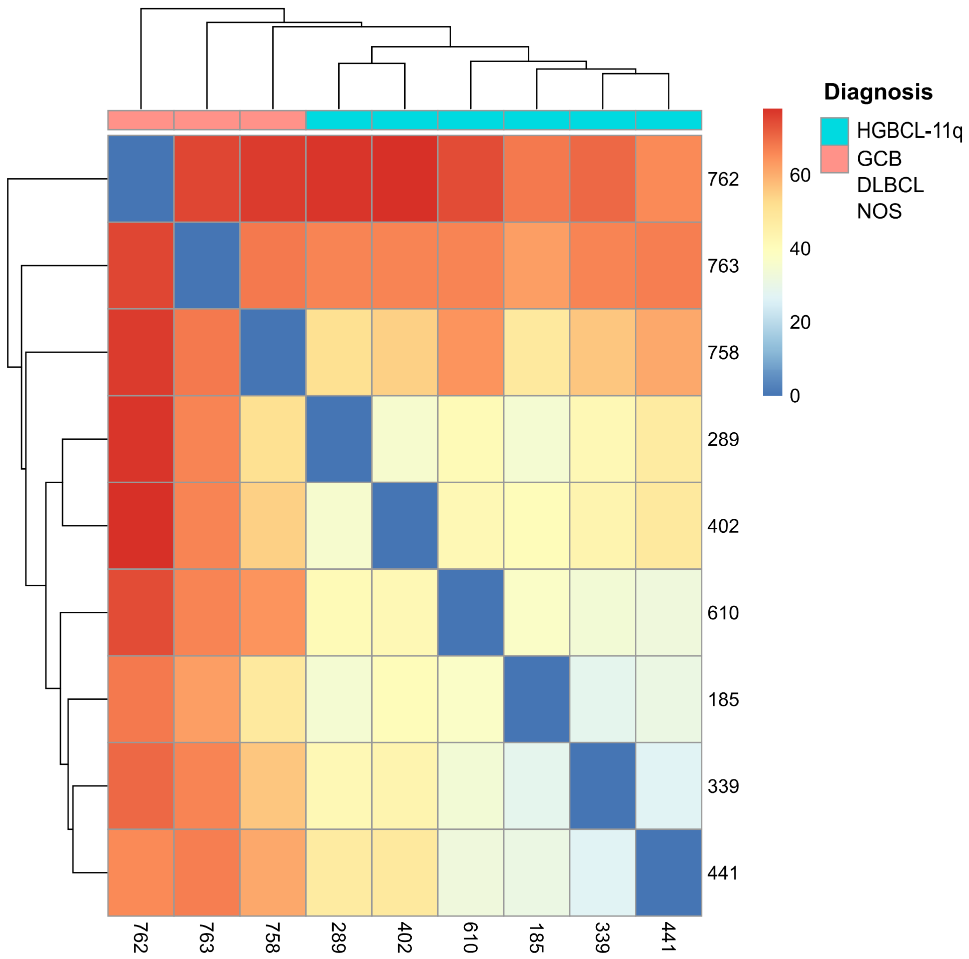

Figure S1. Dendrogram and heatmap illustrating the hierarchical clustering of HGBCL-11q and GCB-DLBCL-NOS without *MYC*-R samples based on the expression levels of all microRNA NGS reads from each individual sample. This analysis highlights the similarities and differences in microRNA expression levels across the individual samples. The colours, as indicated by the scale, represent differences in expression levels, ranging from none (dark blue) to high (dark red).

Multidimensional scaling analysis of HGBCL-11q and GCB-DLBCL-NOS without *MYC*-R microRNA expression profiles

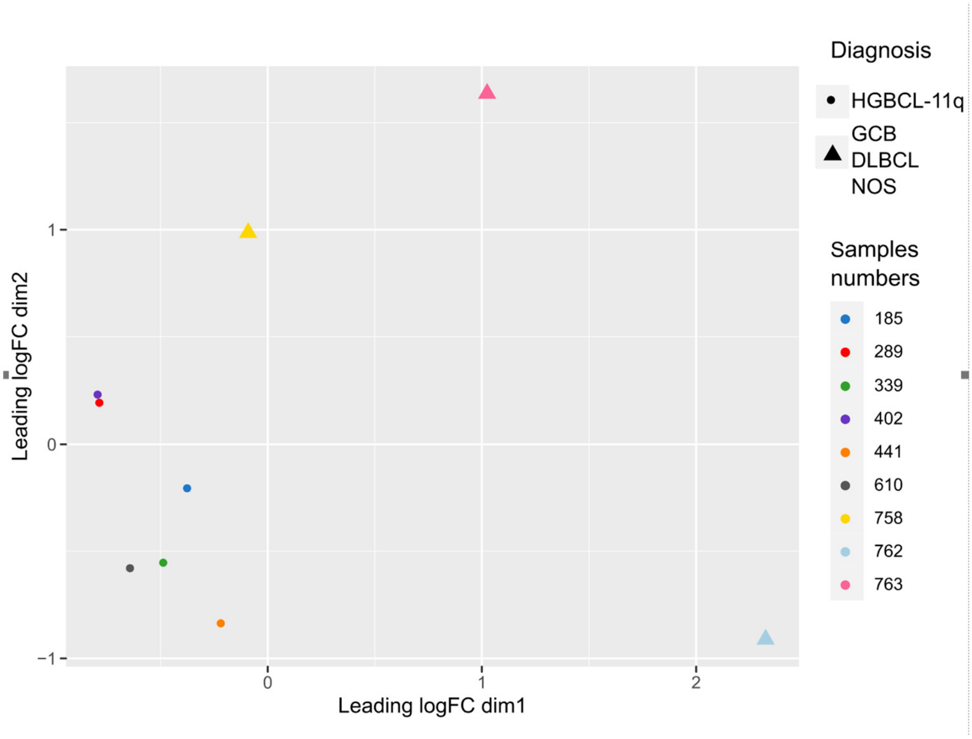

Figure S2. MDS analysis performed for HGBCL-11q and GCB-DLBCL-NOS without *MYC*-R cases based on the expression profiles of NGS reads for all microRNAs. dim1 and dim2 represent the first and second dimensions, equivalents of the first and second principal components in the PCA analysis. The X and Y axes – measure of the diversity of each principal component.

Table S2. microRNAs that expression levels significantly differentiate HGBCL-11q and GCB DLBCL,NOS with *MYC* rearrangement

microRNA	p	*p* after FDR correction	**FC** HGBCL-11q vs. GCB-DLBCL-NOS with *MYC*-R**	Absolute FC value – measure of effect size between HGBCL-11q and. GCB-DLBCL-NOS with *MYC*-R (the direction of change is not determined)
		**Downregulated**		
hsa-miR-3144-3p	0.00168	0.037445	**0.0155**	64.4795
hsa-miR-2115-5p	0.002519	0.048283	**0.0266**	37.6259
**^hsa-miR-193b-3p**	**0.001375**	**0.037005**	**0.0703**	**14.2336**
**^hsa-miR-223-3p**	**1.59E-05**	**0.002434**	**0.072**	**13.8959**
hsa-miR-664a-5p	0.000737	0.023971	**0.0897**	11.1485
hsa-miR-486-5p	0.000626	0.02298	**0.1074**	9.309
hsa-miR-196b-5p	0.001653	0.037445	**0.1406**	7.1108
hsa-let-7b-5p	0.000214	0.011525	**0.1556**	6.4286
hsa-miR-26b-3p	0.001359	0.037005	**0.183**	5.4633
hsa-miR-150-5p	0.002244	0.045975	**0.1846**	5.4167
hsa-let-7c-5p	0.00154	0.037445	**0.1979**	5.052
hsa-miR-361-3p	0.001412	0.037005	**0.2018**	4.9552
hsa-miR-378i	0.001744	0.037445	**0.2207**	4.5306
hsa-let-7g-5p	0.000617	0.02298	**0.3203**	3.1222
hsa-let-7a-5p	0.000162	0.010217	**0.3255**	3.0724
		**Upregulated**		
hsa-miR-3687	5.38×10^{-7}	0.000493	**196.0784**	196.0784
hsa-miR-5585-3p	7.00×10^{-6}	0.001285	**188.6792**	188.6792
hsa-miR-663b	0.000757	0.023971	**87.7193**	87.7193
hsa-miR-3919	0.00258	0.048283	**64.1026**	64.1026
hsa-miR-3648	0.000702	0.023971	**61.7284**	61.7284
hsa-miR-1244	0.00169	0.037445	**49.0196**	49.0196
hsa-miR-1246	2.17×10^{-5}	0.002482	**47.619**	47.619
hsa-miR-5095	0.000128	0.009806	**47.619**	47.619
hsa-miR-3196	0.001728	0.037445	**40.9836**	40.9836
hsa-miR-663a	0.000758	0.023971	**38.1679**	38.1679
hsa-miR-4517	2.85×10^{-5}	0.002744	**37.3134**	37.3134
hsa-miR-619-5p	6.56×10^{-6}	0.001285	**34.965**	34.965
hsa-miR-3960	0.00027	0.013086	**31.4465**	31.4465
hsa-miR-6087	1.40×10^{-6}	0.000495	**30.303**	30.303
hsa-miR-4532	0.001756	0.037445	**29.4985**	29.4985
hsa-miR-1290	0.000167	0.010217	**24.2718**	24.2718
hsa-miR-4466	0.002577	0.048283	**23.2558**	23.2558
hsa-miR-7706	0.000487	0.020313	**20.6186**	20.6186

hsa-miR-1296-3p	0.002353	0.046904	**18.7266**	18.7266
hsa-miR-4488	0.000588	0.02298	**17.1233**	17.1233
hsa-miR-887-3p	0.000164	0.010217	**17.0358**	17.0358
hsa-miR-4423-3p	0.001746	0.037445	**14.1643**	14.1643
hsa-miR-124-3p	0.001409	0.037005	**13.587**	13.587
hsa-miR-301a-5p	0.002256	0.045975	**12.5945**	12.5945
hsa-miR-7974	0.001229	0.036352	**9.4787**	9.4787
hsa-miR-21-3p	0.000202	0.011525	**7.5245**	7.5245
hsa-miR-18a-5p	1.62×10^{-6}	0.000495	**6.6007**	6.6007
hsa-miR-148a-3p	1.89×10^{-5}	0.002475	**5.5463**	5.5463
hsa-miR-142-3p	2.99×10^{-5}	0.002744	**5.3792**	5.3792
hsa-miR-18b-5p	0.001018	0.031123	**4.3879**	4.3879
hsa-miR-19a-3p	0.000321	0.014444	**3.7836**	3.7836
hsa-miR-21-5p	0.000331	0.014444	**3.7736**	3.7736
hsa-miR-19b-3p	7.31×10^{-5}	0.006094	**2.949**	2.949
hsa-miR-16-5p	0.000271	0.013086	**2.4546**	2.4546

- *FDR – false discovery rate
- **FC – fold change; values below 1 - decrease of expression; values above 1 - increase of expression;
- ^ bold green – indicates microRNAs that expression levels differ HGBCL-11q, BL, GCB-DLBCL-NOS and also GCB-DLBCL-NOS *MYC*-R

Dendrogram and heatmap showing the hierarchical clustering of HGBCL-11q and GCB-DLBCL-NOS with *MYC* gene rearrangement

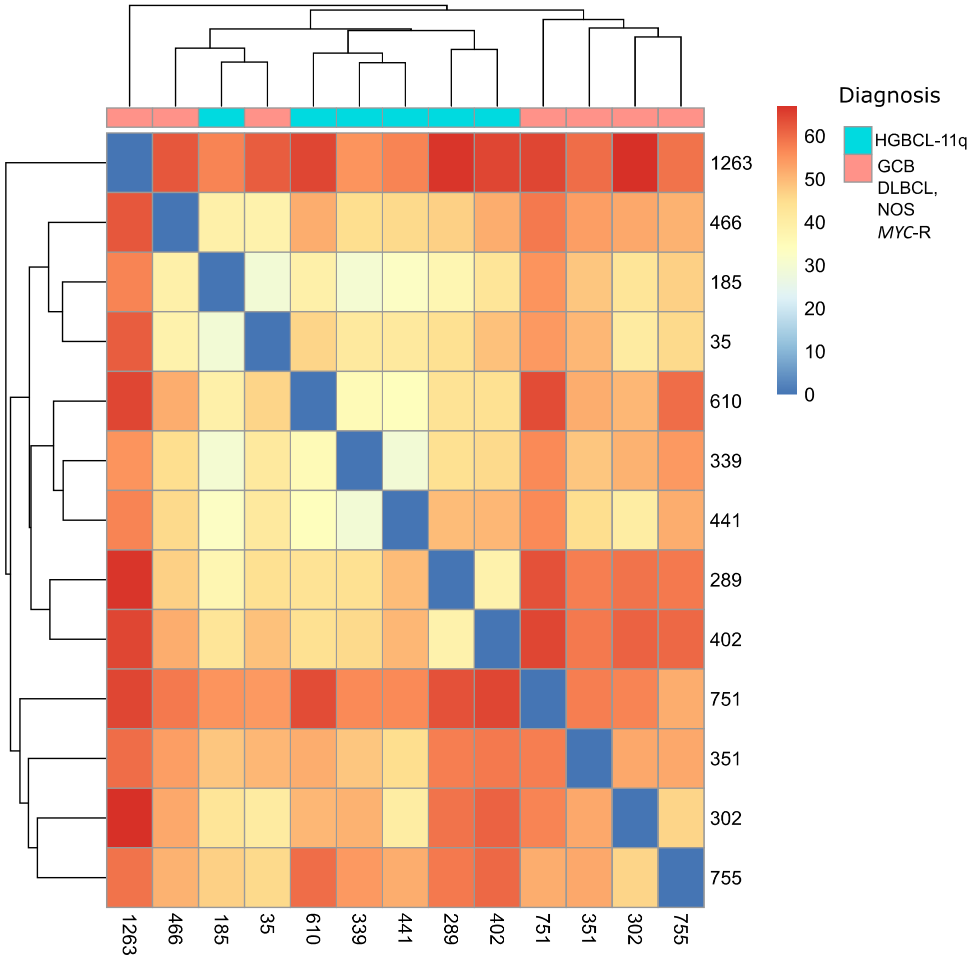

Figure S3. Dendrogram and heatmap showing the hierarchical clustering of HGBCL-11q and GCB-DLBCL-NOS with *MYC* gene rearrangement performed by analysing the expression levels of all microRNA read by NGS for each individual sample. This analysis indicates the similarities and differences in microRNA expression levels across the microRNA expression profile for the individual samples. "0" - dark blue colour indicates no difference in the level of expression; red - the greatest difference in the level of expression; the colour shades illustrate intermediate differences in the level of expression.

MDS analysis performed for HGBCL-11q and GCB-DLBCL-NOS with *MYC* gene rearrangement

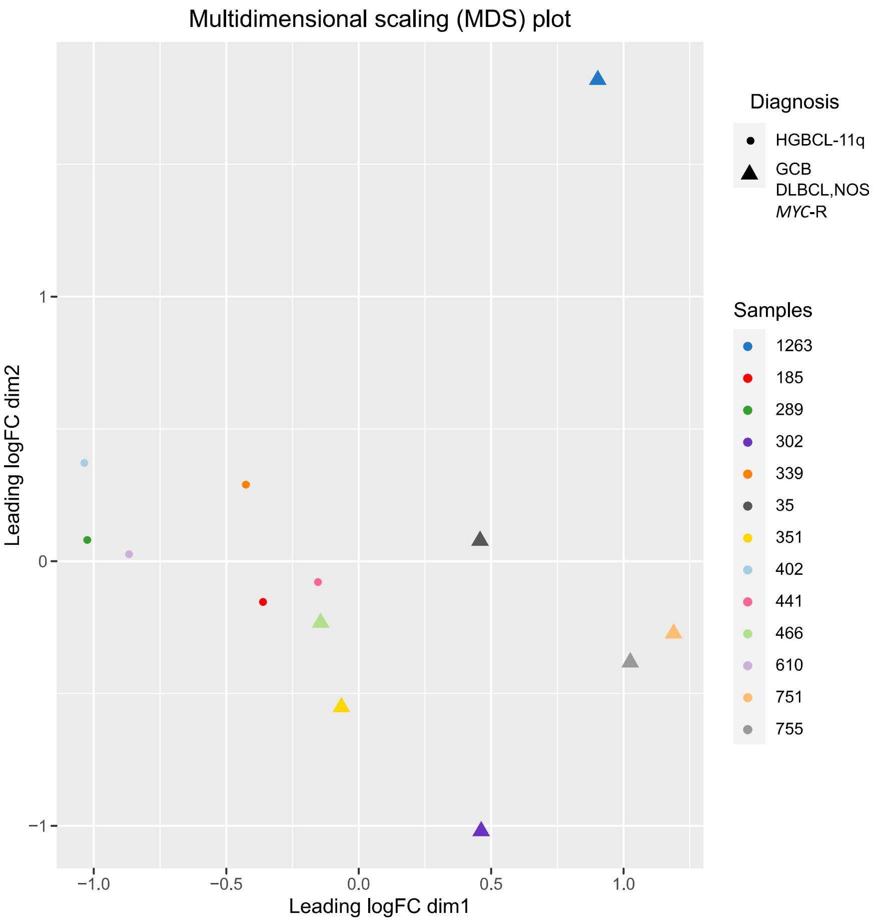

Figure S4. MDS analysis performed for HGBCL-11q and GCB-DLBCL-NOS with *MYC* gene rearrangement cases based on the expression profile of all microRNA read in NGS. dim1, dim2 (abbreviation for dimension) - equivalents of the first and second principal components in the PCA analysis. On the 0X and 0Y axes - measures of the diversity of each principal component.

Dendrogram and heatmap showing the hierarchical clustering of BL and GCB-DLBCL-NOS with *MYC* gene rearrangement

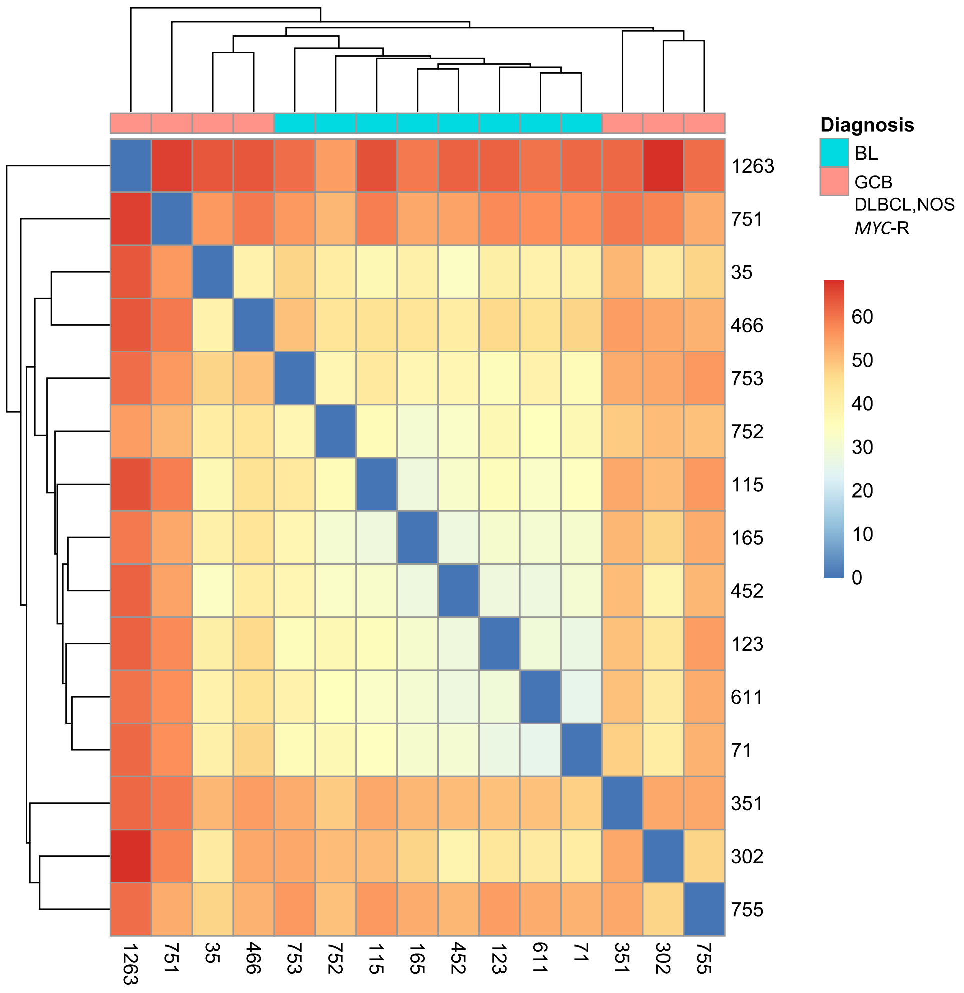

Figure S5. Dendrogram and heatmap showing the hierarchical clustering of BL and GCB-DLBCL-NOS with *MYC* gene rearrangement performed by analysing the expression levels of all microRNA read by NGS for each individual sample. This analysis indicates the similarities and differences in microRNA expression levels across the microRNA expression profile for the individual samples. "0" - dark blue colour indicates no difference in the level of expression; red - the greatest difference in the level of expression; the colour shades illustrate intermediate differences in the level of expression.

MDS analysis performed for BL and GCB-DLBCL-NOS with *MYC* gene rearrangement

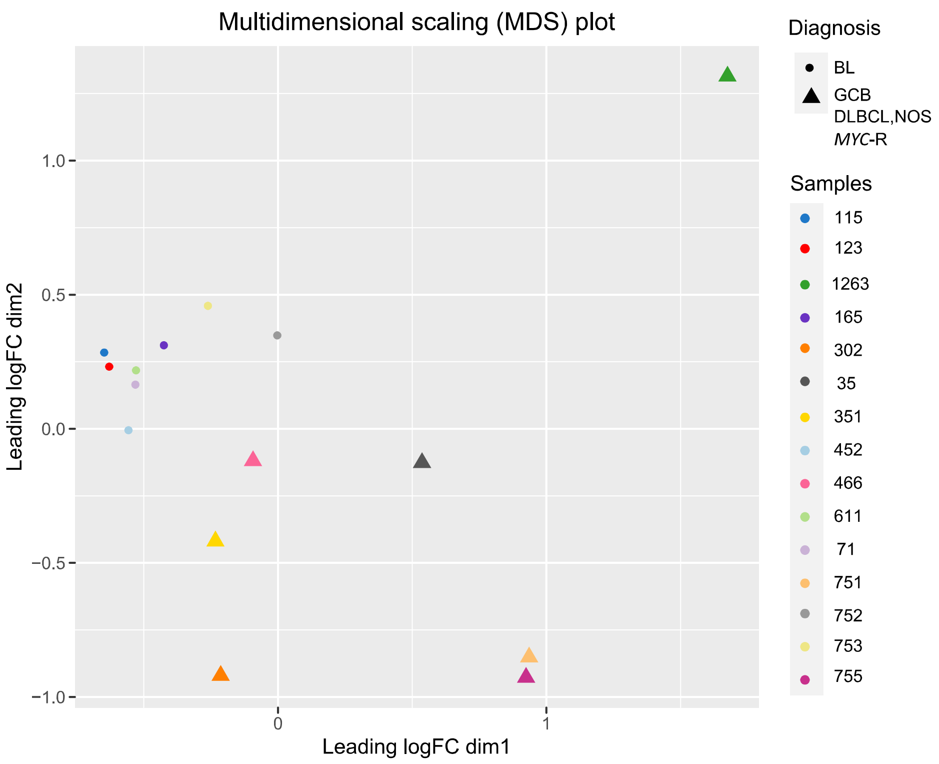

Figure S6. MDS analysis performed for BL and GCB-DLBCL-NOS with *MYC* gene rearrangement cases based on the expression profile of all microRNA read in NGS. dim1, dim2 (abbreviation for dimension) - equivalents of the first and second principal components in the PCA analysis. On the 0X and 0Y axes - measures of the diversity of each principal component.

Table S3: Cytogenetic data of BL and GCB-DLBCL-NOS cases

No.	Karyotype [standard GC-banding - Wright stain] and FISH	Rearrengement		
		MYC	*BCL2*	*BCL6*
	**BL**			
**165**	46,XX,der(1)dup(1)(q25q44)ins(1)(q21q44q25),t(8;14)(q24;q32)[12].<br>nuc ish(BCL6x2)[223],(MYCx2)(5'MYC sep 3'MYCx1)[120/126],(BCL2x2)[214]	(+)<br>95%	(-)	(-)
**71**	46,XY,t(8;22)(q24;q11)[15].<br>nuc ish(BCL6x2)[260],(MYCx2)(5'MYC sep 3'MYCx1)[219],(IGH,BCL2)x2 [259],(IGLx2)(5'IGL sep 3'IGLx1) [229]	(+)<br>100%	(-)	(-)
**123**	46,XY,t(8;14)(q24;q32)[5]/46,idem,i(7)(q10)[10]/46,XY[1].<br>nuc ish(BCL6x2)[257],(MYCx2)(5'MYC sep 3'MYCx1)[164/205]/(5'MYCx2,3'MYCx3)(5'MYC con 3'MYCx1)[21/205],(IGHx3,BCLx2)[91/100]	(+)<br>90%	(-)	(-)
**115**	46,XY,dup(1)(q21q32),t(8;22)(q24;q11)[cp17].<br>nuc ish(MYCx2)(5'MYC sep 3'MYCx1)[102/105],(IGLx2)(5'IGL sep 3'IGLx1)[100/109]	(+)<br>97%	nd	nd
**452**	46,XX,t(8;14)(q24;q32)[13].<br>nuc ish(BCL6x2)[234],(MYCx2)(5'MYC sep 3'MYCx1)[198/215],(IGHx3,BCLx2)[233]	(+)<br>92%	(-)	(-)
**611**	46,XY,t(8;14)(q24;q32)[7]/46,XY[10].<br>nuc ish(MYCx2)(5'MYC sep 3'MYCx1)[37/232],(IGH,BCL2)x2[203]	(+)<br>16%	(-)	nd
**752**	46,XX,t(8;14)(q24;q32)[7]/47,idem,+mar[2]/46,XX[1].<br>nuc ish(BCL6x2)[116],(MYCx2)(5'MYC sep 3'MYCx1)[107/132],(BCL2x2)[117]	(+)<br>81%	(-)	(-)
**753**	45,X,-Y,del(3)(q24q26),t(8;14)(q24;q32),add(11)(q25)[10].<br>nuc ish(BCL6x2)[128],(MYCx2)(3'MYC sep 5'MYCx1)[92/100],(BCL2x2)[107]	(+)<br>92%	(-)	(-)
	**GCB-DLBCL-NOS without *MYC*-R**			
**758**	43~46,XX,del(1)(p36),-2,-3,del(3)(p21),del(6)(q21),+del(6)(q21),add(10)(q22),add(19)(q13),-21,-22,+1~5mar[cp10].<br>nuc ish(BCL6x2)[112],(MYCx2)[107],(BCL2x2)[101]	(-)	(-)	(-)
**762**	no karyotype<br>nuc ish(BCL6x2)[106],(MYCx2)[103],(BCL2x2)[106]	(-)	(-)	(-)
**763**	42~50,XY,+2,+11,+12,+13[cp13].<br>nuc ish(BCL6x2)[101],(MYCx2)[108],(BCL2x2)[103]	(-)	(-)	(-)
	**GCB-DLBCL-NOS with *MYC*-R**			
**466**	51~55,XX,add(1)(p3?6),add(3)(q2?5),del(3)(p2?1p2?5),del(6)(q2?3),-8,-9,add(11)(q2?1),+add(12)(p11.2),+13,-16,+9~19mar[cp10]/46,XX[12].<br>nuc ish(BCL6x3)[7/107],(MYCx2)(5'MYC sep 3'MYCx1)[60/108]	(+)<br>56%	(-)	(-) gain to 3 copies
**351**	41~46,X,-Y,add(1)(p2?6),+der(8)t(8;14)(q24;q32),add(13)(q24),+mar[4]/46,XY[6].<br>nuc ish(BCL6x2)[236],(MYCx3)(5'MYC sep 3'MYCx1)[135/272]/(MYCx2)(5'MYC sep 3'MYCx1) [12/272]/(MYCx3)[15/272],(BCL2x2)[216]	(+)<br>54%	(-)	(-)
**751**	48~49,XX,der(1)(:?:1p36->1q32::1q32->1q21::1q21-->1qter),+add(1)(p22),add(6)(q?27),del(6)(q?15),del(7)(q22)x2,-8,add(14)(q32),+11,+12,+2~3mar[cp9].<br>nuc ish(BCL6x2)[109],(MYCx2)(5'MYC sep 3'MYCx1)[109],(BCL2x2)[108]	(+)<br>100%	(-)	(-)
**755**	76~79,XX,-X,add(1)(p22),+add(1)(p22),-3,del(6)(q23),del(7)(q32),+del(7)(q32),-8,+11,+12,-14,-17,-18,+21,+5~11mar,+r[cp8].<br>nuc ish(BCL6x2)[115],(MYCx4)(5'MYC sep 3'MYCx1)[51/101]/(MYCx3)(5'MYC sep 3'MYCx1)[28/101],(BCL2x3)[52/122]	(+)<br>78%	(-)<br>3 copies in 43%	(-)
**1263**	46,XY,i(1)(q10)[2]/80~85,XXYY,i(1)(q10)x2,-4,-4,del(6)(q23),-10,-13,-14,-14,-15,-19,add(19)(q13)x2,-21,+mar[cp6].<br>nuc ish(BCL6x4)[20/125]/(BCL6x3)[6/125],(5'MYCx3,3'MYCx2)(5'MYC con 3'MYCx2)[24/103]/(5'MYCx6,3'MYCx4)(5'MYC con 3'MYCx4)[6/103]/(5'MYCx4,3'MYCx3)(5'MYC con 3'MYCx3)[6/103],(BCL2x4)[13/130]	(+)<br>23%	(-) gain to 4 copies	(-) gain 3 to 4 copies
**302**	46,XX,+3,t(8;14)(q24;q32),?i(9)(p10),-18[cp12]/46,XX[2].<br>nuc ish(BCL6x3)[15/126]/(BCL6x4)[67/126]/(BCL6x5)[10/126], (MYCx3)(5'MYC sep 3'MYCx1)[82/104],(IGHx4,BCL2x4)[56/101]	(+)<br>52%	(-) gain to 4 copies	(-) gain 3 to 5 copies
**35**	no karyotype<br>nuc ish(BCL6x3)[15/126]/(BCL6x4)[67/126]/.<br>nuc ish(BCL6x5)[10/126],(MYCx3)(5'MYC sep 3'MYCx1)[82/104],(IGH,BCL2)x4[56/101]	(+)<br>82%	(-) gain to 4 copies	(-) gain 3 to 5 copies

No. – sample number; FISH – fluorescence in situ hybridization; add - additional material of unknown origin; cp - composite karyotype; con – connected signals; del – deletion; der - derivative chromosome; dup-duplication; I – isochromosome; idem - denotes the stemline karyotype in a subclone; ins – insertion; mar - marker chromosome; nd – not done; nuc ish - nuclear in situ hybridization; pter – terminal end of the short arm; sdl – sideline; sep - separated signals; sl- stemline; t – translocation.

Table S4: Immunophenotype of HGBCL-11q, BL and GCB-DLBCL-NOS cases (immunohistochemical and flow cytometry data)

No.	CD45	CD20	CD19	CD22	BCL2	CD10	BCL6	CD44	MUM	CD38	^MYC	LMO2	CD56	CD16/56	HLADR	CD79b	CD43	FMC7	CD52	CD71 (%)	Ki67 (%)	KAPPA	LAMBDA	IgD	IgM	IgG	CD62L	CD5	CD11c	CD23	CD25	TdT	EBER	CD49d	CD54	CD81	CD54	CD138	CD200	CD305
**HGBCL-11q**																																								
610	+	+↑	+	+	-	+	+↑	-/+	-	+	+	+/-	+	+	+	nd	+/-	+/-	+	100	100	+	-	-	-	+	-/+	-	-	-	-/+	-	-	nd	nd	nd	nd	+/-↓	nd	nd
339	+	+↑	+	+	-	+	+↑	+/-*	-	+	+/-	+	-	-	+	+	+	+	+	100	100	-	+	-	-/+	+	-	-	-	-	-	-	-	nd	nd	nd	nd	-	nd	nd
289	+	+↑	+	-/+	-	+	+↑	-/+*	-	+	+	-	+/-*	nd	+	+	+/-	-	+	87	100	+	-	-/+	+	-	-/+	-	-	-	-	-	-	nd	nd	nd	nd	+	nd	nd
185.1	+	+↑	+	+	-	+	+↑	+*	+/-	+	-	+	+	+	+	+	+	+	+	100	100	+	-	+	+	-	-	-	-	-	-	-	-	nd	nd	nd	nd	-	nd	nd
441	+↓	+↑	+	+/-	-	+	+↑	+*	-	+	-	+	+*	-	+	+	+/-	+/-	+	100	100	-	-	-	+/-	-	-	-	nd	-	-/+	-	-	nd	nd	nd	nd	-/+↓	-	nd
402	+	+↑	+	+	-	+	+↑	+	-	+	nd	nd	+	+	+	+	+/-	+	+	100	100	-	-	-	+	-	+/-	-	-	-	-	-	-	nd	nd	nd	nd	-	nd	nd
**BL**																																								
165	+	+↑	+	+/-	-	+	+	+	-	+↑	nd	-	-	-	+	nd	+	+	+	100	99	-	+	+	+	-	-/+	-	-	-	+	-	+	nd	nd	nd	nd	+/-	nd	nd
71	+	+↑	+	+	-	+	+	+	-	+↑	+	-	-	-	+	nd	+	+/-	+	100	95	-	+	-	+	-	+/-	-	-/+	-	-	-	-	nd	nd	nd	nd	nd	nd	nd
123	+	+↑	+	+	-	+	nb	+/-	-	+↑	+	-	-	-	+	nd	+	+	+	100	100	+	-	-	+	+/-	-	-	-	-/+	+	-	-	nd	nd	nd	nd	nd	nd	nd
115	+	+↑	+	+/-	-	+	+	-/+	-	+↑	nd	-	-	-	+	+	+	+	nd	100	100	+	-	-	+	-/+	-/+	-	-/+	-	+	-	-	nd	nd	nd	nd	nd	nd	nd
452	+↓	+↑	+	+	-	+	+	+	-	+↑	+	-	-	-	+	nd	+	+	+	100	95	+	-	-	+	+	+	-	-	-	-	-	+	nd	nd	nd	nd	nd	nd	nd
611	+	+↑	+	+	-	+	+	+/-	-	+↑	+	-	-	-	+	nd	+/-	+	+	100	95	+	nd	+/-	+	nd	-	-	-	-	-	-	-	nd	nd	nd	nd	+/-	nd	nd
752	+↓	+↑	+	+	-	+	+	+/-	nd	+↑	nd	nd	nd	-	+	+	+	+	+	100	nd	-	+	+/-	+	-	-	-	+/-	-	+/-	nd	nd	nd	nd	+↑	nd	nd	-	-
753	+↓	+↑	+	+	-	+	+↑	-	-	+↑	+	-	-	-	+	+	+	+	-/+	100	100	-	+	-	+/-	-	-	-	-	-	-	-	+	+	-	+↑	-	-	-	-
**GCB-DLBCL-NOS without *MYC* rearrangement**																																								
758	+↓	+	+	+	+	+/-	+/-	+↑	nd	+	+	-	nd	-	+	+↑	+/-	-	+	100	95	-/+	-	-	-/+	-/+	+↑	-	+/-	-	+/-	nd	-	-	nd	+	nd	nd	-	+/-
762	+↓	+↓	+↓	+	-*	+	+	-	-/+	+	+	+↑	+/-	-	+↑	-	+	-	-	100	80	-	-	-	+	-	-/+	-	+	-	-	nd	-	-/+	+↑	+↓	+↑	nd	-	+↑
763	+↓	+	+	+	-	-	+	+	nd	+	-	nd	-	-	+/-	+	+	-	+	100	90	-	-	-	+	+	+/-	-	-/+	-	+	-	-	-	+↑	+	+↑	-	-	+
**GCB-DLBCL-NOS with *MYC* rearrangement**																																								
466	+↓	+↑	+	+	-	+	+	+	-	+↑	nd	+↑	nd	-	+	-*	+/–	+	+↑	100	90	-	-/+	-	+/-	-	+	–	-/+	+/-	-	-	-	nd	nd	nd	nd	nd	+/-	nd
351	+↓	+↑	+	+	+↑	+	nd	+/-	nd	+	nd	nd	+	nd	+	+	-/+	+	nd	62	nd	-	-	-	+/-	+/-	+/-	-	-/+	-	nd	nd	nd	nd	nd	nd	nd	nd	nd	nd
751	+↓	-/+	+	-/+	+	+	-	+*	+/-	+↑	+↑	-	-	-	+	+/-	+*	-	+/-	52	100	-	-	-	+	+	-	-	-	-	+/-	nd	-	nd	nd	+	nd	-	-	-/+
755	+↑	+↑	+↓	+↑	+	+	+	+	-	+↑	nd	nd	nd	-	+	+↑	+↓	-	+↑	100	80	-	-	+	+	+	nd	-	-	-	+/-	nd	nd	-	+↑	+↑	+↑	nd	-	+
1263	+	+↑	+	+	-/+	+	+↑	+	-	+	+	+	-/+	-/+	+	+	+↓	+	+	100	>95	+	-	+	-/+	-	-	-	+	-	-	nd	-	nd	+↑	+	+↑	-	-	-
302	+↑	+	+	+	-	+	nd	-	nd	+	nd	nd	nd	-	+	nd	-	+	+	100	nd	+	-	-	-/+	+	-	-	-/+	-	-	nd	nd	nd	nd	nd	nd	+	nd	nd
35	+	+	+	+	-	+	+	+	nd	+	+	+↑	+	-	+	nd	+/-	+	-	100	95	nd	-	-	-	-	+	-	-	-/+	-	nd	-	nd	nd	nd	nd	+	nd	nd

No. – sample number; [–] – expression in <20% of neoplastic cells (blue); [–/+] – expression in >20%<50% of neoplastic cells (green) and [+/–] – expression in >50%<100% of neoplastic cells (green); [+] – expression in 100% of neoplastic cells (red); [+↑] – expression higher (dark red when stronger); or [+↓] – expression weaker in lymphoma cells, compared to normal B/T lymphocytes (bright red); * small differences in immunohistochemical and flow cytometry; ^MYC- positive expression when seen in more than 40% of lymphoma cells; nd – not done (grey).

Immunohistochemistry and flow cytometry analyses were performed as previously described in Rymkiewicz G et al., 2018 [14] in Supplementary Appendix

Table S5: List of cytogenetic probes

Probe name	Description	Cat. No.	Manufacturer
CEP 11 (D11Z1)	SpectrumAqua Probe, Centromeric probe	06J54-011	
LSI CCND1	Break Apart Rearrangement Probe	05N38-020	
LSI ATM (11q22.3)	SpectrumOrange Probe, Copy Number Probe	01N33-020	
LSI MLL	Dual Color, Break Apart Rearrangement Prot	08L57-020	Vysis Abbott Molecular, Downer Grove, IL
TelVysion 11q D11S103[illegible]	SpectrumOrange, Copy Number Probe	05J04-011	
LSI MYC	Dual Color Break Apart Rearrangement	05J91-001	
LSI BCL2	Dual Color Break Apart Rearrangement Prob	05N51-020	
LSI BCL6	Dual Color Break Apart Rearrangement Prob	01N23-020	

Reference microRNAs

The results obtained in qPCR for microRNAs chosen as potentially reference molecules, were analysed in *NormFinder_0953* (add-on for Exel) to assess their stability (Table S6).

Table S6. The expression stability assessment of potentially reference microRNAs

microRNA	Value that define the microRNA expression stability – the lower the higher stability
miR-26a-5p	0,084
miR-148b-3p	0,088
miR-103a-3p	0,098
miR-185-5p	0,115
miR-26b-5p	0,142

The qPCR results were calculated in relation to arithmetic mean of all five above mentioned microRNAs, because all met the criteria of the stability analysis were satisfactory for all selected microRNAs

Verification of the microRNA NGS results performed on HGBCL-11q and BL groups

The verification of microRNA sequencing was performed using RT-qPCR technique. The verification confirmed NGS results. The expression level of microRNAs: miR-155-5p, miR-21-5p, miR-29b-2-5p, miR-29b-3p and miR-200c-3p were higher in HGBCL,11q than in BL. The expression level of miR-223-3p and miR-1295a were lower in HGBCL,11q than in BL. All results were statistically significant (Figure S7).

Verification of microRNA sequencing using RT-qPCR technique

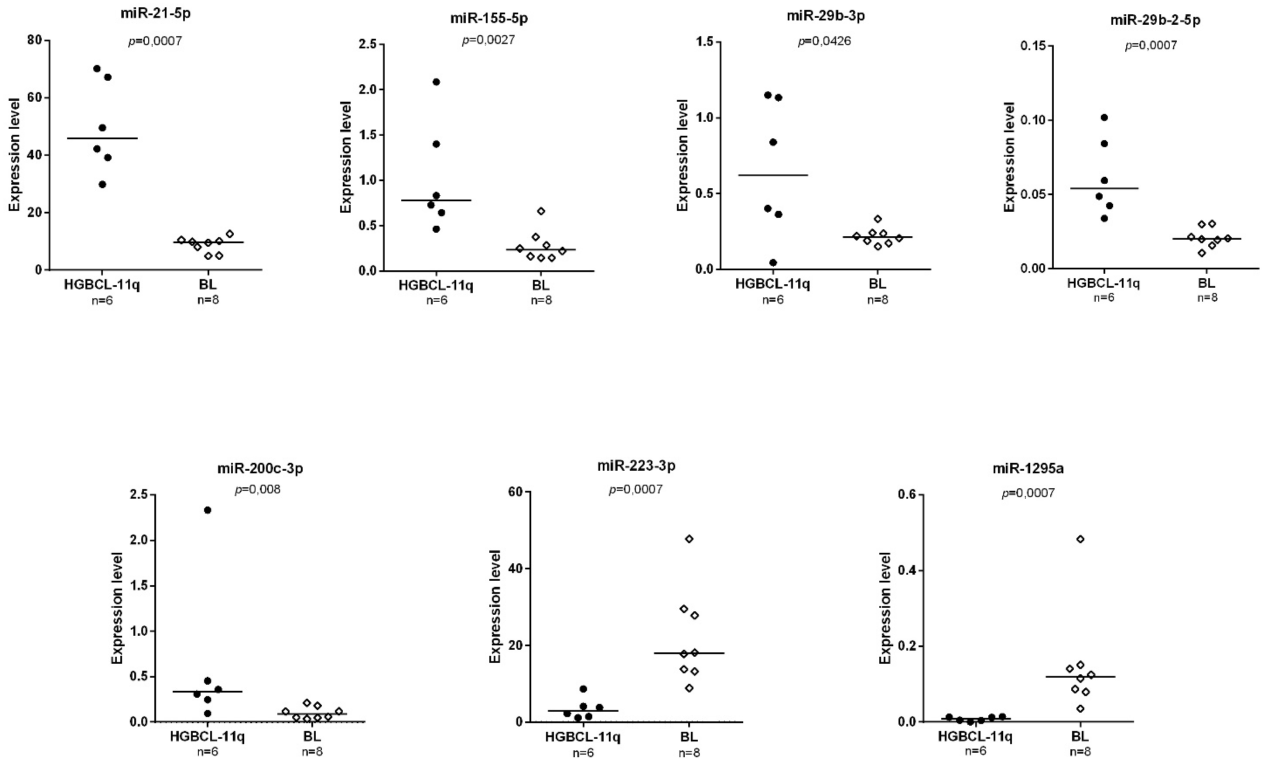

Figure S7. Assessment of miR-155-5p, miR-21-5p, miR-29b-2-5p, miR-29b-3p, miR-200c-3p miR-223-3p and miR-1295a expression levels in HGBCL,11q and in BL performed using RT-qPCR

Supplement 2

The results of microRNA expression profiles ontological analysis in HGBCL-11q and BL

Table S7. The most highly expressed signalling pathways and/or processes involving microRNA that expression levels significantly differentiate HGBCL-11q and BL. The result obtained using the miEAA programme and the databases: Reactome and miRPathDB

**Pathway/ reaction**	***padj***	**microRNA**
Interleukin-4 and Interleukin-13 signaling	0.000169	miR-21-5p; miR-200c-3p; miR-3919; miR-34c-5p; miR-223-3p; miR-663a; miR-29b-3p; miR-203a-3p; miR-155-5p; miR-184; miR-146a-5p; miR-34a-3p
Estrogen-dependent nuclear events downstream of ESR-membrane signaling	0.000768	miR-21-5p; miR-200c-3p; miR-34c-5p; miR-4464; miR-29b-3p; miR-155-5p; miR-184; miR-146a-5p; miR-34a-3p
Signal Transduction	0.000837	miR-21-5p; miR-200c-3p; miR-34c-5p; miR-223-3p; miR-663a; miR-29b-3p; miR-203a-3p; miR-155-5p; miR-184; miR-146a-5p; miR-34a-3p; miR-193b-3p
Extra-nuclear estrogen signaling	0.001437	miR-21-5p; miR-200c-3p; miR-34c-5p; miR-29b-3p; miR-203a-3p; miR-34b-5p; miR-155-5p; miR-184; miR-34a-3p
RUNX3 regulates WNT signaling	0.001437	miR-200c-3p; miR-3919; miR-34c-5p; miR-34b-5p; miR-34a-3p; miR-193b-3p
Gene expression Transcription	0.003818	miR-21-5p; miR-21-3p; miR-200c-3p; miR-34c-5p; miR-223-3p; miR-4464; miR-29b-3p; miR-203a-3p; miR-34b-5p; miR-155-5p; miR-146a-3p; miR-184; miR-146a-5p; miR-34a-3p; miR-30d-5p; miR-193b-3p
AKT phosphorylates targets in the nucleus	0.003909	miR-21-5p; miR-223-3p; miR-29b-3p; miR-184
Deactivation of the beta-catenin transactivating complex	0.003909	miR-200c-3p; miR-34b-5p; miR-155-5p; miR-184; miR-34a-3p; miR-193b-3p
ESR-mediated signaling	0.003909	miR-21-5p; miR-200c-3p; miR-3919; miR-34c-5p; miR-29b-3p; miR-34b-5p; miR-155-5p; miR-146a-3p; miR-184; miR-34a-3p; miR-193b-3p

Table S7 continued. The most highly expressed signalling pathways and/or processes involving microRNA that expression levels significantly differentiate HGBCL-11q and BL. The result obtained using the miEAA programme and the databases: Reactome and miRPathDB

**Pathway/ reaction**	***padj***	**microRNA**
Generic Transcription Pathway	0.003909	miR-21-5p; miR-21-3p; miR-200c-3p; miR-34c-5p; miR-223-3p; miR-4464; miR-29b-3p; miR-203a-3p; miR-34b-5p; miR-155-5p; miR-146a-3p; miR-184; miR-146a-5p; miR-34a-3p; miR-193b-3p
Interleukin-10 signaling	0.003909	miR-21-5p; miR-223-3p; miR-146a-5p
SUMO E3 ligases SUMOylate target proteins	0.003909	miR-21-5p; miR-200c-3p; miR-223-3p; miR-29b-3p; miR-155-5p; miR-34a-3p; miR-193b-3p
SUMOylation	0.003909	miR-21-5p; miR-200c-3p; miR-223-3p; miR-29b-3p; miR-155-5p; miR-34a-3p; miR-193b-3p
Signaling by cytosolic FGFR1 fusion mutants	0.003909	miR-21-5p; miR-223-3p; miR-203a-3p; miR-155-5p
Cellular Senescence	0.004533	miR-21-5p; miR-200c-3p; miR-34c-5p; miR-223-3p; miR-29b-3p; miR-203a-3p; miR-155-5p; miR-146a-3p; miR-146a-5p; miR-30d-5p; miR-193b-3p
Signaling by Receptor Tyrosine Kinases	0.004533	miR-21-5p; miR-200c-3p; miR-34c-5p; miR-223-3p; miR-29b-3p; miR-203a-3p; miR-155-5p; miR-146a-5p
Interleukin-6 signaling	0.005073	miR-223-3p; miR-203a-3p; miR-155-5p; miR-146a-5p
Cytokine Signaling in Immune system	0.008624	miR-21-5p; miR-200c-3p; miR-3919; miR-223-3p; miR-29b-3p; miR-203a-3p; miR-155-5p; miR-184; miR-146a-5p
FGFR1 mutant receptor activation	0.00896	miR-21-5p; miR-223-3p; miR-203a-3p; miR-155-5p
Senescence-Associated Secretory Phenotype SASP	0.00896	miR-200c-3p; miR-34c-5p; miR-223-3p; miR-29b-3p; miR-155-5p; miR-146a-3p; miR-146a-5p
Signaling by FGFR1 in disease	0.00896	miR-21-5p; miR-200c-3p; miR-223-3p; miR-203a-3p; miR-155-5p
Signaling by Interleukins	0.00896	miR-21-5p; miR-200c-3p; miR-34c-5p; miR-223-3p; miR-29b-3p; miR-203a-3p; miR-155-5p; miR-184; miR-146a-5p

Table S8. Twenty biological processes/pathways regulated by microRNA that expression levels significantly differentiate HGBCL-11q and BL. The result obtained using the miEAA programme and the databases: Gene Ontology and miRPathDB

**Biological processes (/pathways)**	***padj***	**microRNA**
aging	7.452 x 10^{-6}	miR-21-5p; miR-200c-3p; miR-223-3p; miR-663a; miR-29b-3p; miR-1908-5p; miR-203a-3p; miR-155-5p; miR-146a-5p; miR-34a-3p; miR-193b-3p
positive regulation of cell activation	7.452 x 10^{-6}	miR-21-5p; miR-200c-3p; miR-34c-5p; miR-223-3p; miR-29b-3p; miR-34b-5p; miR-155-5p; miR-184; miR-146a-5p
positive regulation of fibroblast proliferation	7.452 x 10^{-6}	miR-21-5p; miR-200c-3p; miR-34c-5p; miR-29b-3p; miR-203a-3p; miR-34b-5p; miR-184; miR-146a-5p; miR-193b-3p
regulation of chromatin organization	7.452 x 10^{-6}	miR-21-5p; miR-200c-3p; miR-34c-5p; miR-223-3p; miR-663a; miR-29b-3p; miR-203a-3p; miR-155-5p; miR-34a-3p; miR-30d-5p
regulation of ossification	7.452 x $10^{-6\ 6}$	miR-21-5p; miR-200c-3p; miR-34c-5p; miR-223-3p; miR-29b-3p; miR-203a-3p; miR-34b-5p; miR-155-5p; miR-146a-5p; miR-34a-3p
cellular response to oxidative stress	9.759 x 10^{-6}	miR-21-5p; miR-200c-3p; miR-34c-5p; miR-223-3p; miR-29b-3p; miR-203a-3p; miR-155-5p; miR-184; miR-34a-3p; miR-193b-3p
regulation of mesenchymal cell proliferation	9.759 x 10^{-6}	miR-21-5p; miR-200c-3p; miR-223-3p; miR-29b-3p; miR-203a-3p; miR-34b-5p; miR-155-5p; miR-34a-3p
cell development	9.821 x 10^{-6}	miR-21-5p; miR-200c-3p; miR-34c-5p; miR-223-3p; miR-663a; miR-29b-3p; miR-203a-3p; miR-155-5p; miR-184; miR-146a-5p; miR-34a-3p; miR-30d-5p
fibroblast proliferation	9.821 x 10^{-6}	miR-21-5p; miR-200c-3p; miR-34c-5p; miR-663a; miR-29b-3p; miR-34b-5p; miR-155-5p; miR-146a-5p; miR-34a-3p
locomotion	9.821 x 10^{-6}	miR-21-5p; miR-200c-3p; miR-34c-5p; miR-223-3p; miR-663a; miR-29b-3p; miR-203a-3p; miR-155-5p; miR-184; miR-146a-5p; miR-34a-3p
ossification	9.821 x 10^{-6}	miR-21-5p; miR-200c-3p; miR-34c-5p; miR-223-3p; miR-29b-3p; miR-203a-3p; miR-155-5p; miR-184; miR-146a-5p; miR-34a-3p
regulation of fibroblast proliferation	9.821 x10^{-6}	miR-21-5p; miR-200c-3p; miR-34c-5p; miR-663a; miR-29b-3p; miR-34b-5p; miR-155-5p; miR-146a-5p; miR-34a-3p
cell migration	1.01 x 10^{-5}	miR-21-5p; miR-200c-3p; miR-34c-5p; miR-223-3p; miR-663a; miR-29b-3p; miR-203a-3p; miR-155-5p; miR-184; miR-146a-5p; miR-34a-3p
mesenchymal cell proliferation	1.373 x 10^{-5}	miR-21-5p; miR-200c-3p; miR-29b-3p; miR-203a-3p; miR-34b-5p; miR-155-5p; miR-34a-3p
regulation of intracellular signal transduction	1.822 x 10^{-5}	miR-21-5p; miR-200c-3p; miR-34c-5p; miR-223-3p; miR-663a; miR-29b-3p; miR-203a-3p; miR-155-5p; miR-184; miR-146a-5p; miR-34a-3p; miR-193b-3p

Table S8 continued. Twenty biological processes/pathways regulated by microRNA that expression levels significantly differentiate HGBCL-11q and BL. The result obtained using the miEAA programme and the databases: Gene Ontology and miRPathDB

**Biological processes (/pathways)**	***padj***	**microRNA**
cell motility	1.913×10^{-5}	miR-21-5p; miR-200c-3p; miR-34c-5p; miR-223-3p; miR-663a; miR-29b-3p; miR-203a-3p; miR-155-5p; miR-184; miR-146a-5p; miR-34a-3p
response to radiation	1.92×10^{-5}	miR-21-5p; miR-223-3p; miR-663a; miR-29b-3p; miR-155-5p; miR-184; miR-146a-5p; miR-34a-3p; miR-193b-3p
head development	2.493×10^{-5}	miR-21-5p; miR-200c-3p; miR-34c-5p; miR-223-3p; miR-663a; miR-29b-3p; miR-203a-3p; miR-34b-5p; miR-155-5p; miR-146a-5p
positive regulation of cell population proliferation	2.536×10^{-5}	miR-21-5p; miR-200c-3p; miR-34c-5p; miR-223-3p; miR-4464; miR-29b-3p; miR-203a-3p; miR-34b-5p; miR-155-5p; miR-184; miR-146a-5p; miR-34a-3p
BMP signaling pathway	2.763×10^{-5}	miR-200c-3p; miR-34c-5p; miR-203a-3p; miR-155-5p; miR-146a-5p; miR-34a-3p

Table S9. The most highly expressed molecular functions in which are involved microRNAs with significantly higher expression levels in HGBCL-11q compared to BL (analysis performed using miEAA and Gene Ontology & miRPathDB)

**Molecular function**	***padj***	**microRNA**
DNA-binding transcription activator activity RNA polymerase II-specific	5.97 x 10^{-5}	miR-21-5p; miR-200c-3p; miR-34c-5p; miR-223-3p; miR-663a; miR-29b-3p; miR-203a-3p; miR-34b-5p; miR-155-5p; miR-28-5p; miR-30d-5p
identical protein binding	5.97 x 10^{-5}	miR-21-5p; miR-223-3p; miR-29b-3p; miR-203a-3p; miR-155-5p; miR-184; miR-146a-5p; miR-193b-3p
protein dimerization activity	5.97 x 10^{-5}	miR-21-5p; miR-34c-5p; miR-223-3p; miR-29b-3p; miR-155-5p; miR-146a-3p
molecular function regulator	6.46 x 10^{-5}	miR-21-5p; miR-200c-3p; miR-34c-5p; miR-223-3p; miR-29b-3p; miR-203a-3p
cytokine receptor binding	0.000549	miR-21-5p; miR-223-3p; miR-29b-3p; miR-203a-3p; miR-146a-5p
signaling receptor binding	0.00082	miR-21-5p; miR-200c-3p; miR-223-3p; miR-29b-3p; miR-203a-3p; miR-146a-5p
cyclin binding	0.001046	miR-34c-5p; miR-223-3p; miR-203a-3p; miR-34b-5p; miR-155-5p; miR-193b-3p
RNA polymerase II-specific DNA-binding transcription factor binding	0.002584	miR-21-5p; miR-200c-3p; miR-203a-3p; miR-34b-5p; miR-155-5p; miR-146a-5p
signaling receptor activator activity	0.002757	miR-223-3p; miR-29b-3p; miR-146a-5p
histone acetyltransferase binding	0.002806	miR-21-5p; miR-200c-3p; miR-223-3p; miR-155-5p
catalytic activity	0.003002	miR-21-5p; miR-200c-3p; miR-29b-3p; miR-203a-3p; miR-155-5p; miR-146a-5p; miR-193b-3p
SMAD binding	0.003312	miR-21-5p; miR-200c-3p; miR-374b-3p; miR-29b-3p; miR-203a-3p; miR-34b-5p; miR-34a-3p
double-stranded DNA binding	0.003312	miR-21-5p; miR-200c-3p; miR-34c-5p; miR-223-3p; miR-29b-3p; miR-203a-3p; miR-34b-5p; miR-155-5p; miR-28-5p; miR-146a-5p; miR-30d-5p
protein C-terminus binding	0.003312	miR-21-5p; miR-200c-3p; miR-663a; miR-203a-3p
transcription factor binding	0.003312	miR-21-5p; miR-200c-3p; miR-223-3p; miR-29b-3p; miR-203a-3p; miR-34b-5p; miR-155-5p;

		miR-34a-3p; miR-193b-3p

Table S9 continued. The most highly expressed molecular functions in which are involved microRNAs with significantly higher expression levels in HGBCL-11q compared to BL (analysis performed using miEAA and Gene Ontology & miRPathDB)

**Molecular function**	***padj***	**microRNA**
transcription factor binding	0.003312	miR-21-5p; miR-200c-3p; miR-223-3p; miR-29b-3p; miR-203a-3p; miR-34b-5p; miR-155-5p; miR-34a-3p; miR-193b-3p
transforming growth factor beta receptor cytoplasmic mediator activity	0.003312	miR-34c-5p; miR-203a-3p; miR-155-5p; miR-146a-5p; miR-34a-3p
transcription regulatory region DNA binding	0.004459	miR-21-5p; miR-200c-3p; miR-34c-5p; miR-223-3p; miR-29b-3p; miR-203a-3p; miR-34b-5p; miR-155-5p; miR-28-5p; miR-146a-5p; miR-30d-5p
enzyme binding	0.004938	miR-21-5p; miR-200c-3p; miR-34c-5p; miR-223-3p; miR-29b-3p; miR-203a-3p; miR-155-5p; miR-146a-5p; miR-30d-5p; miR-193b-3p
RNA polymerase II proximal promoter sequence-specific DNA binding	0.005389	miR-21-5p; miR-200c-3p; miR-34c-5p; miR-223-3p; miR-29b-3p; miR-203a-3p; miR-155-5p; miR-30d-5p
heat shock protein binding	0.005389	miR-200c-3p; miR-34c-5p; miR-155-5p; miR-193b-3p
lipid kinase activity	0.005389	miR-21-5p; miR-663a; miR-29b-3p; miR-155-5p
chromatin binding	0.005389	miR-21-5p; miR-200c-3p; miR-223-3p; miR-29b-3p; miR-203a-3p; miR-155-5p; miR-193b-3p

Table S10. Twenty of the most highly expressed biological processes (/ ontology terms) regulated by microRNAs that expression levels significantly differentiate HGBCL-11q and BL groups (analysis performed using miEAA and databases: Gene Ontology and miRTarBase)

**Biological processes/ ontology terms**	***padj***	**microRNA**
B cell proliferation GO0042100	0.0020789	miR-21-5p; miR-200c-3p; miR-3919; miR-34c-5p; miR-5585-3p; miR-223-3p; miR-4464; miR-29b-3p; miR-34b-5p; miR-184; miR-146a-5p; miR-34a-3p; miR-30d-5p; miR-193b-3p
arginine catabolic process GO0006527	0.003098	miR-21-5p; miR-200c-3p; miR-29b-2-5p; miR-374b-3p; miR-155-5p; miR-146a-5p; miR-3681-3p; miR-30d-5p; miR-193b-3p
E-box binding GO0070888	0.009426	miR-21-5p; miR-21-3p; miR-200c-3p; miR-3919; miR-34c-5p; miR-619-5p; miR-223-3p; miR-663a; miR-3922-5p; miR-4423-3p; miR-29b-2-5p; miR-29b-3p; miR-1908-5p; miR-203a-3p; miR-34b-5p; miR-155-5p; miR-28-3p; miR-184; miR-28-5p; miR-1258; miR-3681-3p; miR-34a-3p; miR-30d-5p; miR-193b-3p
basal plasma membrane GO0009925	0.0109032	miR-21-5p; miR-1295a; miR-200c-3p; miR-34c-5p; miR-619-5p; miR-223-3p; miR-663a; miR-4464; miR-29b-2-5p; miR-1908-5p; miR-34b-5p; miR-155-5p; miR-146a-3p; miR-184; miR-2116-5p; miR-28-5p; miR-146a-5p; miR-34a-3p; miR-30d-5p; miR-193b-3p
catenin complex GO0016342	0.0109032	miR-21-5p; miR-200c-3p; miR-34c-5p; miR-663a; miR-4464; miR-374b-3p; miR-1908-5p; miR-203a-3p; miR-34b-5p; miR-155-5p; miR-663b; miR-2116-5p; miR-3648; miR-3681-3p; miR-34a-3p; miR-193b-3p
cell aging GO0007569	0.0109032	miR-21-5p; miR-21-3p; miR-200c-3p; miR-619-5p; miR-223-3p; miR-663a; miR-4464; miR-4423-3p; miR-29b-3p; miR-155-5p; miR-28-3p; miR-28-5p; miR-3681-3p; miR-34a-3p; miR-30d-5p; miR-193b-3p
cellular response to indole-3-methanol GO0071681	0.0109032	miR-21-5p; miR-34c-5p; miR-203a-3p; miR-34b-5p; miR-155-5p; miR-146a-5p; miR-34a-3p; miR-193b-3p
cellular response to prostaglandin D stimulus GO0071799	0.0109032	miR-21-5p; miR-155-5p; miR-193b-3p
channel inhibitor activity GO0016248	0.0109032	miR-21-5p; miR-200c-3p; miR-34c-5p; miR-29b-3p; miR-34b-5p; miR-184; miR-34a-3p
copper ion binding GO0005507	0.0109032	miR-21-5p; miR-34c-5p; miR-619-5p; miR-223-3p; miR-663a; miR-3922-5p; miR-29b-2-5p; miR-29b-3p; miR-203a-3p; miR-155-5p; miR-28-3p; miR-28-5p; miR-146a-5p;

		miR-3681-3p; miR-30d-5p; miR-221-5p; miR-193b-3p

Table S10 continued. Twenty of the most highly expressed biological processes (/ ontology terms) regulated by microRNAs that expression levels significantly differentiate HGBCL-11q and BL groups (analysis performed using miEAA and databases: Gene Ontology and miRTarBase)

**Biological processes/ ontology terms**	***padj***	**microRNA**
dendrite arborization GO0140059	0.0109032	miR-223-3p; miR-155-5p; miR-193b-3p
copper ion binding GO0005507	0.0109032	miR-21-5p; miR-34c-5p; miR-619-5p; miR-223-3p; miR-663a; miR-3922-5p; miR-29b-2-5p; miR-29b-3p; miR-203a-3p; miR-155-5p; miR-28-3p; miR-28-5p; miR-146a-5p; miR-3681-3p; miR-30d-5p; miR-221-5p; miR-193b-3p
dendrite arborization GO0140059	0.0109032	miR-223-3p; miR-155-5p; miR-193b-3p
fibroblast apoptotic process GO0044346	0.0109032	miR-21-5p; miR-3919; miR-34c-5p; miR-29b-3p; miR-34b-5p; miR-155-5p; miR-184; miR-34a-3p; miR-30d-5p
humoral immune response GO0006959	0.0109032	miR-21-5p; miR-200c-3p; miR-3919; miR-34c-5p; miR-5585-3p; miR-619-5p; miR-223-3p; miR-29b-2-5p; miR-29b-3p; miR-203a-3p; miR-34b-5p; miR-155-5p; miR-184; miR-146a-5p; miR-3681-3p; miR-34a-3p; miR-30d-5p; miR-193b-3p
negative regulation of cellular pH reduction GO0032848	0.0109032	miR-21-5p; miR-200c-3p; miR-34c-5p; miR-29b-3p; miR-34b-5p; miR-184; miR-34a-3p
ossification involved in bone remodeling GO0043932	0.0109032	miR-21-5p; miR-663a; miR-29b-3p; miR-155-5p; miR-146a-5p
positive regulation of T-helper cell differentiation GO0045624	0.0109032	miR-200c-3p; miR-34c-5p; miR-34b-5p; miR-155-5p; miR-193b-3p
positive regulation of metanephric cap mesenchymal cell proliferation GO0090096	0.0109032	miR-21-5p; miR-3919; miR-34c-5p; miR-29b-3p; miR-34b-5p; miR-155-5p; miR-184; miR-34a-3p; miR-30d-5p
regulation of cell cycle arrest GO0071156	0.0109032	miR-21-5p; miR-619-5p; miR-4423-3p; miR-29b-2-5p; miR-29b-3p; miR-203a-3p; miR-155-5p; miR-663b; miR-184; miR-2116-5p; miR-3681-3p; miR-34a-3p; miR-193b-3p
regulation of mitochondrial membrane permeability GO0046902	0.0109032	miR-21-5p; miR-200c-3p; miR-3919; miR-34c-5p; miR-223-3p; miR-663a; miR-29b-3p; miR-34b-5p; miR-28-3p; miR-184; miR-2116-5p; miR-28-5p; miR-146a-5p; miR-3681-3p; miR-34a-3p; miR-30d-5p; miR-

		221-5p; miR-193b-3p
regulation of somatic stem cell population maintenance GO1904672	0.0109032	miR-21-5p; miR-3919; miR-34c-5p; miR-29b-3p; miR-34b-5p; miR-155-5p; miR-184; miR-34a-3p; miR-30d-5p
